# Cytotoxicity-Guided Isolation of Two New Phenolic Derivatives from *Dryopteris fragrans* (L.) Schott

**DOI:** 10.3390/molecules23071652

**Published:** 2018-07-06

**Authors:** Tong Zhang, Li Wang, De-Hua Duan, Yi-Hao Zhang, Sheng-Xiong Huang, Ying Chang

**Affiliations:** 1 College of Life Science, Northeast Agricultural University, Harbin 150030, China; shmzhyzt@163.com (T.Z.); myrddh@163.com (D.-H.D.); yhzneau@163.com (Y.-H.Z.); 2 State Key Laboratory of Phytochemistry and Plant Resources in West China, Kunming Institute of Botany, Chinese Academy of Sciences, Kunming 650201, China; jasminewangli@126.com

**Keywords:** cytotoxicity-guided, phenolic derivatives, *Dryopteris fragrans*, chemical derivatization, immuno-regulation activity

## Abstract

*Dryopteris fragrans* is a valuable medicinal plant resource with extensive biological activities including anti-cancer, anti-oxidation, and anti-inflammation activities. This work aims to study further the cytotoxic constituents from *Dryopteris fragrans*. In this work, two new phenolic derivatives known as dryofragone (**1**) and dryofracoumarin B (**2**) with six known compounds (**3**–**8**) were isolated from the petroleum ether fraction of the methanol extract of the aerial parts of *Dryopteris fragrans* (L.) Schott by two round cytotoxicity-guided tracking with the 3-(4,5-dimethyl-2-thiazolyl)-2,5-diphenyl-2-*H*-tetrazolium bromide (MTT) assay and cell counting kit-8 (CCK-8) assay. Their structures were elucidated by the extensive spectroscopic analysis (^1^H-NMR, ^13^C-NMR, and two dimensions NMR), chemical derivatization, and comparison with data reported in the literature. All the isolates were evaluated for their cytotoxicity against nine cancer cell lines as well as their in vitro immunomodulatory activity. The results showed that compounds have a modest cytotoxicity toward human HeLa cell line with IC_50_ value below 30 μM and compounds **4** and **5** may modulate immunity to affect the growth of tumor cells.

## 1. Introduction

**Figure 1:**
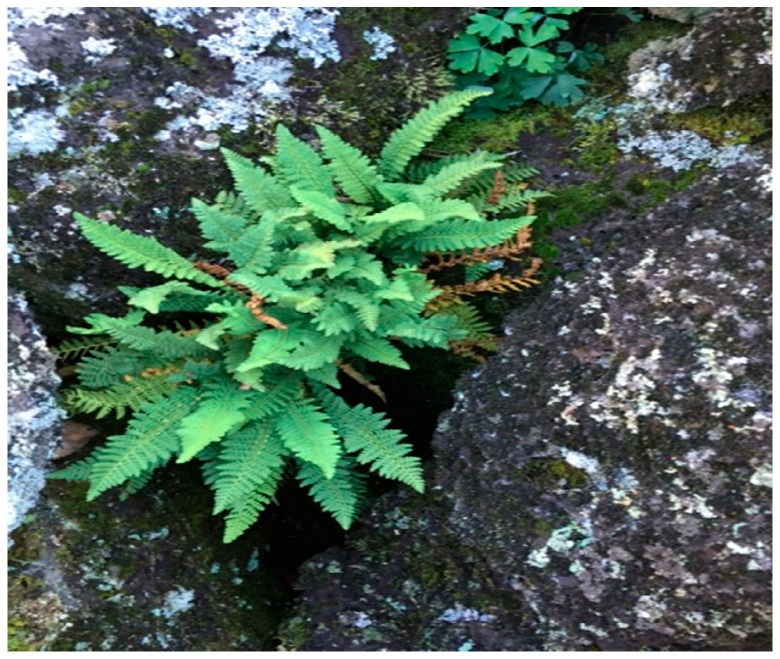
*Dryopteris fragrans* plant.

*Dryopteris fragrans* (L.) Schott (Figure 1) belonging to the genus Dryopteris is a perennial herb with aroma widely distributed throughout the world and is mostly distributed in the alpine and volcanic regions of Northeast China [1,2]. *D. fragrans* has been used as folk medicine for treating arthritis and skin diseases such as psoriasis, dermatophytosis, and more [3]. Previous phytochemical investigations on this plant have led to the identification of terpenoids [4], phloroglucinols [5], glucosides [6], and other phenolic derivatives such as coumarin [3]. The earlier biological studies have shown that *D. fragrans* was a valuable medicinal plant resource with extensive biological activities including anti-cancer, anti-oxidation, insect repellent, anti-microbial, and anti-inflammation activities [3,4,5,6,7].

Of its various biological effects, the mechanism of anti-cancer effects has been studied most. Dryofragin, which is a derivative of phloroglucinol, was found to activate the endogenous pathway of apoptosis by affecting the changes of ROS in mitochondria and inducing changes in mitochondria in breast cancer cell MCF-7 and to cause tumor cell apoptosis by the apoptosis-related protein Bcl-2, Bax, Caspase-9, Caspase-3, and PARP [8]. It has also been reported to be an inhibitor of migration and invasion of the human osteosarcoma cell line U2OS through the PI3K/Akt and MAPK energy pathway involving MMP-2/9 and TIMP-1/2 proteins [9]. Aspidin PB, which is another phloroglucinol derivative from *D. fragrans*, has been recorded as a tumor cell-inhibiting agent for its impact on cyclin p53/p21 and mitochondrial changes in human osteosarcoma cells Saos-2, U2OS, and HOS [10]. In addition, there have been many other reports on compounds from *D. fragrans* with cytotoxicity [11,12,13]. To further study cytotoxic constituents from *D. fragrans*, a cytotoxicity-guided isolation of the extract of *D. fragrans* was designed. The isolation of two new phenolic derivatives and six known compounds by cytotoxicity-guided tracking as well as their cytotoxicity and immunomodulatory activity detection is described in this paper.

## 2. Results and Discussion

### 2.1. Determination of Isolated Compounds

**Figure 2:**
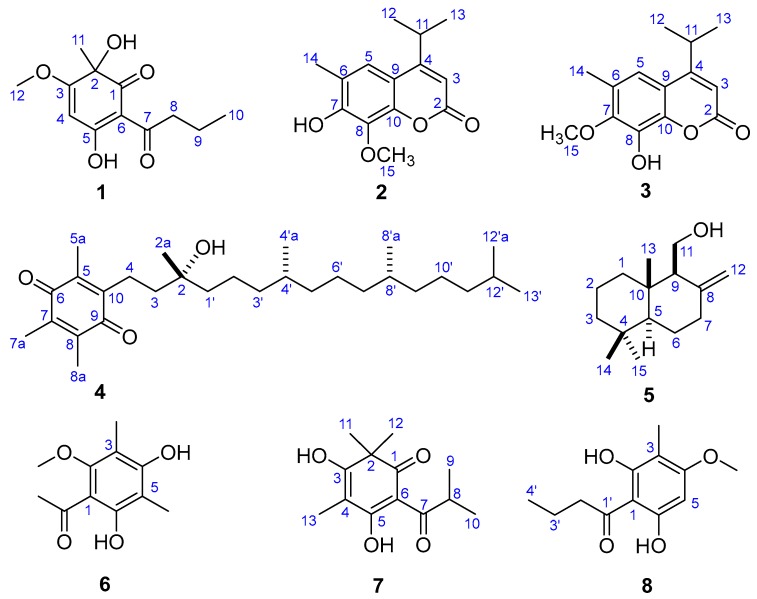
Structures of compounds **1**–**8**.

After two round cytotoxicity screening by MTT [14] and CCK-8 [15] assay, Fractions SG1‒SG7 from the petroleum ether-soluble part with prominent cytotoxic activities were selected as the bioactive sites (Figures S1 and S2). Two new phenolic derivatives known as dryofragone (**1**) and dryofracoumarin B (**2**) (Figure 2) along with six known compounds (**3**–**8**) (Figure 2), were isolated from the above seven bioactive fractions by using extensive chromatographic methods like silica gel, MCI gel, Sephadex LH-20, and HPLC. The known compounds were identified as dryofracoumarin A (**3**) [3], vitamin E quinone (**4**) [16], albicanol (**5**) [5], 2′,4′-dihydroxy-6′-methoxy-3′,5′-dimethylchalcone (**6**) [17], norflavesone (**7**) [18], and aspidinol (**8**) [19] by comparing their ^1^H- and ^13^C-NMR data with that reported in the literature.

**Figure 3:**
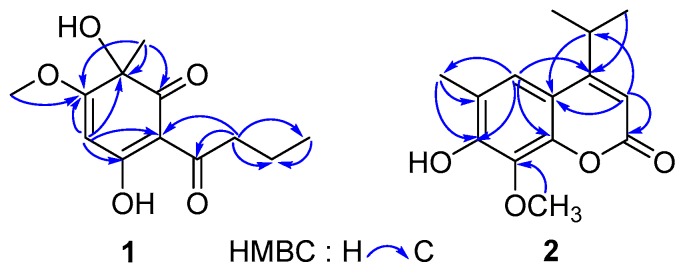
Key HMBC correlations of **1** and **2**.

Compound **1** was obtained as yellow powder from CHCl_3_. The HR-ESI-MS data (*m*/*z* 239.0926 [M − H]^−^, calcd for 239.0925) of **1** showed the molecular formula C_12_H_16_O_5_, which correspond to five degrees of unsaturation. The IR spectrum of **1** displayed hydroxyls (3321 cm^−1^), carbonyl groups (1714 cm^−1^), and double bonds (1607 cm^−1^) absorptions. The red shifted hydroxyl signal (3321 cm^−1^) also showed that some hydroxyls in **1** were involved in the hydrogen bonding interaction. The ^1^H-NMR spectrum of **1** (Table 1) showed one 3H-singlet at *δ*_H_ 1.54 for a tertiary methyl group, one 3H-singlet at *δ*_H_ 3.91 for a methoxy group, one 3H-triplet at *δ*_H_ 1.01 for a primary methyl group, and an olefinic proton at *δ*_H_ 5.37. The ^13^C-NMR spectrum of **1** revealed 12 resonance signals including two ketone carbons at *δ*_C_ 196.3 (conjugated) and 203.7, two pair of olefinic carbons (*δ*_C_ 189.4, 176.0, 104.5 and 94.5) with two oxygenated sites (*δ*_C_ 189.4 and 176.0), an oxygenated tertiary carbon (*δ*_C_ 75.4), a methoxy carbon (*δ*_C_ 57.4), two aliphatic methylene carbon (*δ*_C_ 41.0 and 18.7), and two methyl carbons (*δ*_C_ 30.2 and 14.1). The above evidence indicated that compound **1** presumably possessed an oxygenated phloroglucinol core [20]. This inference was further confirmed by the 2D-NMR spectra (Figure 3). The long-range HMBC couplings—H-4/C-2, C-3, C-5, and C-6 as well as Me-11/C-1, C-2, and C-3 demonstrated the presence of a cyclohexadiene moiety with two oxygen-bearing carbon at C-2 and C-5. The HMBC correlation from a methoxy at *δ*_H_ 3.91 (Me-12) to a quaternary olefinic carbon at *δ*_C_ 176.0 (C-3) revealed that a methoxy was located at C-3. Furthermore, a butyryl was linked to C-6, which was supported by the HMBC correlations from protons at C-8 (*δ*_H_ 2.99 and 2.92) to carbons at *δ*_C_ 104.5 (C-6), 203.7 (C-7), 18.7 (C-9), and 14.1 (C-10), respectively. The CD experiment towards compound **1** was performed. However, the CD spectrum (Figure S9) of **1** showed no characteristic cotton effect. Compound **1** was considered to be a pair of enantiomers.

Therefore, the structure of **1** was concluded to be a new acylphloroglucinol, 6-isobutyryl-2,5-dihydroxy-2-methyl-3-methoxy-cyclohexa-3,5-dien-1-one, and was named dryofragone.

**Figure 4:**
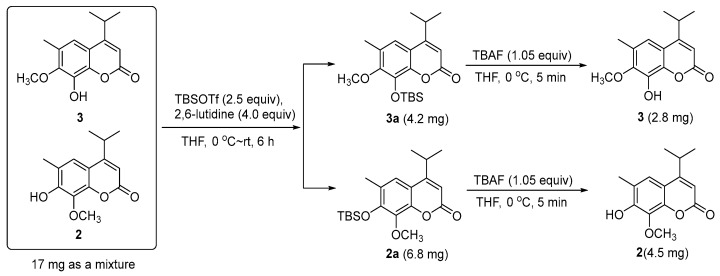
Silicon etherification involved the isolation of **2** and **3**.

Compound **2** was obtained as a mixture with compound **3** initially. The ^13^C-NMR spectrum of the mixture revealed 28 resonance signals (Figure S12) in which half were consistent with the data reported for a coumarin and dryofracoumarin A (**3**) [3]. However, the ESI-MS data (*m*/*z* 249[M + H]^+^, 271[M + Na]^+^, 287[M + K]^+^) of the mixture showed only one molecular weight (248 Da), which aligned with that of **3**. Consequently, the other half of carbon resonance signals in the ^13^C-NMR for the mixture, which were highly similar with that of **3**, were supposed to be of an isomer of **3** featuring exchanged positions of hydroxyl and methoxy groups in the coumarin core. Based on the large space size of tert-butyl dimethyl silicyl group, which can strike the balance of molecular polarity for compounds **2** and **3** and the high yield of the desilication step, a silicon etherification-desilication procedure was designed for the isolation of the mixture (See Section 3.5 and Figure 4). NMR data of compounds **2** and **3** are shown in Table 2. After the chemical derivatization, compound **2** was afforded as a simplex. The IR spectrum of **2** exhibited a signal of hydroxyl with no hydrogen bonds (3548 cm^−1^), a strong band at 1668 cm^−1^ for the lactone subunit in coumarin core, and absorptions (1636, 1602, 1572 cm^−1^) of benzene ring moiety in coumarin. The HR-ESI-MS data (*m*/*z* 247.0975 [M − H]^−^, calcd for 247.0976) indicated a molecular formula C_14_H_16_O_4_ with seven degrees of unsaturation for **2**. The HMBC correlations (Figure 3) from Me-12 and -13 (*δ*_H_ 1.30 × 2) to C-4 (*δ*_C_ 163.0) as well as the correlations from H-11(*δ*_H_ 3.25) to C-3 (*δ*_C_ 107.8), C-4 (*δ*_C_ 163.0), and C-9 (*δ*_C_ 112.2), which suggests that an isopropyl was fused to C-4. Another HMBC correlation Me-15/C-8 verified that a methoxyl group was linked to C-8. In addition, the HMBC correlations from an isolated methyl (*δ*_H_ 2.31) to C-5 (*δ*_C_ 120.0), C-6 (*δ*_C_ 121.1), and C-7 (*δ*_C_ 150.0) inferred a methyl at C-6 in **2**. The above analyses disclosed our former hypothesis. As a result, the structure of **2** was determined to be 7-hydroxy-6-methyl-8-methoxy-4-isopropyl-2H-chromen-2-one, which was given the trivial name of dryofracoumarin B.

### 2.2. In Vitro Cytotoxicity and Immunomodulatory Activity Detection

**Figure 5:**
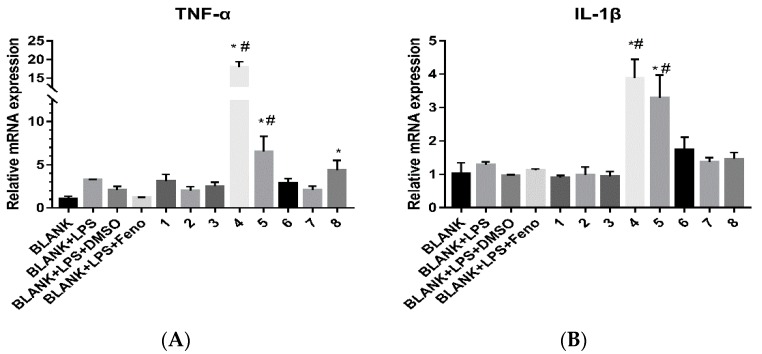
The influence of eight compounds on immuno-regulation factors over a period of 24 h. (**A**) for the factor *TNF-α* and (**B**) for the factor *IL-1β*. The “*” indicates that there were significant differences (*p* < 0.05) between other compounds and the BLANK group amd the “#” indicates that there were significant differences (*p* < 0.05) between other compounds and the BLANK + LPS group. Each value represented the means ± SD of three independent experiments.

For all the isolates, their cytotoxicities against nine human cancer cell lines known as HepG2, A549, HeLa, U251, HOS, MG63, U2OS, MB231, and SKBR-3 as well as their immuno-regulation activities were evaluated. The cytotoxicities were screened using the CCK-8 assay [15]. The IC_50_ values of cytotoxicities for the eight compounds are shown in Table 3. For compounds isolated by cytotoxicity-guided tracking, they exhibited moderate activities to the HeLa cell line and weak activities to glioma, liver cancer, and lung cancer cell lines. However, they were not very sensitive to osteosarcoma and breast cancer cell lines when compared to the crude extract. For their immuno-regulation activities, LPS stimulated THP-1 cells were used as the in vitro model for the detection [21]. Fenofibrate (Feno) pre-treatment (20 μM) was used as a positive control [21]. The results for immuno-regulation activities are shown in Figure 5. Only compounds **4** and **5** could enhance the secretion of the factors *TNF-α* and *IL-1β*. The results showed that compounds **4** and **5** may activate the LPS signaling pathway, which may modulate immunity to affect the growth of tumor cells.

## 3. Materials and Methods

### 3.1. General Experimental Procedures

Optical rotations were recorded in MeOH using a JASCO P-1020 Polarimeter (Jasco Corp., Tokyo, Japan). UV spectra were acquired in MeOH with a Shimadzu UV-2401PC UV-VIS spectrophotometer (Shimadzu Corp., Kyoto, Japan). IR spectra were measured on a Bruker Tensor 27 FTIR Spectrometer with KBr disks (Bruker Corp., Karlsruhe, Germany). ^1^H-NMR, ^13^C-NMR, and 2D NMR spectra were recorded in CDCl_3_ using a Bruker AVANCE III-600 spectrometer or a Bruker DRX-400 spectrometer (Bruker Corp., St. Gallen, Switzerland). TMS was used as the internal standard. ESI-MS spectra were recorded using a Waters Xevo TQ-S Ultra High Pressure Liquid Chromatography Triple Quadrupole Mass Spectrometer (Waters Corp., Manchester, UK). HR-ESI-MS data were obtained using an Agilent G6230 Q-TOF mass instrument (Agilent Corp., Santa Clara, CA, USA). Column chromatography (CC) was performed using a silica gel (200–300 mesh, Qingdao Marine Chemical Inc., Qingdao, China), MCI gel CHP 20P (75–150 µm, Mitsubishi Corp., Tokyo, Japan), and Sephadex LH-20 (25–100 mm, Pharmacia Biotech Ltd., Uppsala, Sweden). Thin-layer chromatography (TLC) was performed using pre-coated silica gel GF254 plates (0.25 mm in thickness for analysis and 0.60 mm thickness for preparation, Qingdao Marine Chemical Inc., Qingdao, China) with various solvent systems. Spots were visualized under UV light (254 nm) and colored by iodine and by spraying silica gel plates with 10% H_2_SO_4_ in MeOH followed by heating. Preparative HPLC separations were performed on a CXTH system equipped with a UV3000 detector (Beijing Chuangxintongheng Instruments Co. Ltd., Beijing, China), and a Kromasil C_18_ column (250 mm × 20 mm i.d., 5 mm, EKA Chemicals Corp., Bohus, Sweden) using a flow rate of 8.0 mL/min at a column temperature of 25 °C. Semi-preparative HPLC was conducted on a HITACHI Chromaster system (Hitachi Ltd., Tokyo, Japan) equipped with an Agilent ZORBAXSB-C_18_ column (150 mm × 9.4 mm i.d., 5 mm, Agilent Corp., Santa Clara, CA, USA) using a flow rate of 3.0 mL/min at a column temperature of 25 °C. The detection was performed with a DAD detector.

### 3.2. Plant Material

The aerial parts of *Dryopteris fragrans* (L.) Schott were collected in June 2016 from the Wudalianchi scenic area, Heihe City, Helongjiang Province, China and identified by Prof. Baodong Liu from the Harbin Normal University. A voucher specimen (No. df-20070702-9) was deposited in the Plant Herbarium of Northeast Agricultural University in Harbin, China.

### 3.3. Determination of Anti-Tumor Fraction of Dryopteris fragrans

After methanol extraction, the crude extract was then partitioned with petroleum ether (rt), dichloromethane (DCM) (rt), EtOAc (rt), and n-BuOH (rt) in sequence. The crude extract was divided into six parts (the whole extracts, petroleum ether layer, DCM layer, EtOAc layer, n-BuOH layer, and water phase). Each fraction was dissolved in DMSO and the final concentration of DMSO in the cell culture medium was no more than 0.1%. The osteosarcoma cell lines HOS and MG63 were used as the first round screening target in MTT [14] for the above six parts of the crude extracts. As shown in Figure S1, petroleum ether fraction of crude extracts had the most obvious cytotoxic effects at the point of 48 h. The petroleum ether fraction was then divided into 14 sub-fractions (Fr SG1‒SG14) by using silica gel column chromatography. The above 14 fractions were then subjected to MTT or CCK-8 assay [15] against HepG2, MB231, and MG63 cell lines, respectively. As shown in Figure S2, Fractions SG1–SG7 from the petroleum ether-soluble part exhibited prominent cytotoxic activities.

### 3.4. Extraction and Isolation

The air-dried aerial parts of *Dryopteris fragrans* (L.) Schott powder (2 kg) were extracted with 100% methanol (20 L × 2 d × 3) and ultrasonized (40 Hz) for 4 h at each time. After filtration, the filtrate was concentrated to yield the crude extract. The crude extract was then suspended in water (1.5 L) and partitioned with petroleum ether (3 × 1.5 L), DCM (3 × 1.5 L), EtOAc (3 × 1.5 L), and n-BuOH (3 × 1.5 L) sequentially. Guided by the first round cytotoxicity screening, the petroleum ether fraction (54 g) was chosen for further isolation. The petroleum ether-soluble part was then subjected to silica gel CC and eluted with petroleum ether–EtOAc (1:0–0:1) to create 14 fractions (SG1–SG14). According to the second round cytotoxicity screening, Fractions SG1–SG7 were selected as the isolation targets for the next step. Fractions SG1 and SG2 were not actually involved in the next step because their low polarities made an effective separation on column chromatography difficult.

Fraction SG3 (5.93 g) was submitted to the silica gel CC (petroleum ether–EtOAc 1:0–0:1) and Sephadex LH-20 CC (MeOH–CHCl_3_ 1:1) and followed by preparative TLC (petroleum ether–EtOAc 11:2, Rf = 0.53) to afford compound **4** (14.4 mg). Compound **5** (103.2 mg) was isolated from Fraction SG4 (5.01 g) by undergoing a protocol of repeated silica gel CC (petroleum ether–EtOAc 1:0–10:1), Sephadex LH-20 CC (MeOH–CHCl3 1:1), and preparative TLC (petroleum ether–EtOAc 8:1, Rf = 0.40). Fraction SG6 (3.54 g) was chromatographed on MCI CC (MeOH–H_2_O 40:60 to 100:0) to yield 16 sub-fractions (Fr M1–M16). Further purification of Fr. M6 by semi-preparative HPLC (MeOH: H_2_O 53:47) resulted in the isolation of compounds **6** (15.8 mg) and **7** (10.0 mg). Fraction SG7 (2.49 g) was further separated by MCI CC (MeOH–H_2_O 20:80 to 100:0) to yield 11 fractions (Fr. M_2_1–M_2_11). Compound **1** (4.5 mg) was purified from Fraction M_2_4 using semi-preparative HPLC (MeOH:H_2_O 58:42). In the same way, compound **8** (2.0 mg) was obtained from Fraction M_2_7. Compounds **2** and **3** were obtained as a mixture (17.0 mg) from Fraction M_2_6 by semi-preparative HPLC (MeOH:H_2_O 77:23). They were separated by a silicon etherification-desilication procedure (See Section 3.5).

Dryofragone (**1**): yellow powder (CHCl_3_). [*α*${]}_{D}^{23.7}$ –20.3 (*c* 0.10, MeOH); UV (MeOH) *λ*_max_ (log *ε*): 198 (3.17) nm, 241 (3.38) nm, 276 (3.20) nm, 320 (3.14) nm, IR (KBr) *ν*_max_ IR (KBr) *ν*_max_ 3321, 2929, 1714, 1607, 1533, 1442, 1231, 1104; ^1^H-NMR (600 MHz, CDCl_3_): *δ*_H_ 5.37 (1H, s, H-4), 3.91 (3H, s, Me-12), 2.99 (1H, m, H-8a), 2.92 (1H ,m, H-8b), 1.69 (2H, m, H-9), 1.54 (3H, s, Me-11), 1.01 (3H, t, *J* = 7.4 Hz, Me-10); 203.7 (C-7), 196.3 (C-1), 189.4 (C-5), 176.0 (C-3), 104.5 (C-6), 94.5 (C-4), 75.4 (C-2), 57.4 (C-12), 41.0 (C-8), 30.2 (C-11), 18.7 (C-9),14.1 (C-10), ESI-MS *m*/*z* 239 [M − H]^−^, and HR-ESI-MS *m*/*z* 239.0926 [M − H]^−^ (calcd for C_12_H_15_O_5_, 239.0925).

### 3.5. Silicon Etherification Involved Isolation of 2 and 3

#### 3.5.1. Silicon Etherification of the Mixture of Compounds **2** and **3**

With regard to the solution of the mixture of Compounds **2** and **3** (17 mg, 0.068 mmol) in dry DCM (0.5 mL), 2,6-luditine (30 μL, 0.27 mmol, 4.0 equiv) was added at 0 °C, which is followed by the addition of TBSOTf (35 μL, 0.17 mmol, 2.5 equiv). The resulting mixture was warmed to room temperature (rt) naturally, stirred for 6 h, and then quenched with water (2.0 mL). The mixture was then stirred for 10 min, followed by an extraction with EtOAc (10.0 mL) three times, and the EtOAc layer was dried over anhydrous Na_2_SO_4_ and subsequently concentrated. The residue was further purified by semi-preparative HPLC (85% MeOH in H_2_O, 3 mL/min, a HITACHI Chromaster system equipped with a DAD detector, an Agilent ZORBAX SB-C_18_ column, 150 mm × 9.4 mm i.d., 5 μm) to yield compound **2a** (6.8 mg, *t*_R_ = 19.8 min) and **3a** (4.2 mg, *t*_R_ = 15.8 min) as white solids. Compound **2a**: ^1^H-NMR (400 MHz, CDCl_3_): *δ*_H_ 7.17 (1H, s, H-5), 6.18 (1H, s, H-3), 3.91 (3H, s, Me-15), 3.24 (1H, m, H-11), 2.29 (3H, s, Me-14), 1.30 (6H, d, *J* = 6.8 Hz, Me-12, 13), 1.03 (9H, s, Me-19, 20, 21), 0.24 (6H, s, Me-16, 17), ^13^C-NMR (100 MHz, CDCl_3_): *δ*_C_ 162.5 (C-4), 161.7 (C-2), 150.1 (C-7), 146.9 (C-10), 138.3 (C-8), 126.5 (C-6), 119.5 (C-5), 113.4 (C-9), 108.5 (C-3), 61.1 (C-15), 28.7 (C-11), 26.1 × 3 (C-19, 20, 21), 22.1 × 2 (C-12, 13), 19.0 (C-18), 17.7 (C-14), −4.0 × 2 (C-16, 17). Compound **3a**: ^1^H-NMR (400 MHz, CDCl_3_): *δ*_H_ 7.07 (1H, s, H-5), 6.21 (1H, s, H-3), 3.82 (3H, s, Me-15), 3.24 (1H, m, H-11), 2.32 (3H, s, Me-14), 1.30 (6H, d, *J* = 6.8 Hz, Me-12, 13), 1.08 (9H, s, Me-19, 20, 21), 0.25 (6H, s, Me-16, 17), ^13^C-NMR (100 MHz, CDCl_3_): *δ*_C_ 162.2 (C-4), 161.4 (C-2), 152.5 (C-7), 145.3 (C-10), 137.2 (C-8), 127.8 (C-6), 117.0 (C-5), 115.4 (C-9), 109.8 (C-3), 60.2 (C-15), 28.7 (C-11), 25.9 × 3 (C-19, 20, 21), 22.1 × 2 (C-12, 13), 18.8 (C-18), 16.4 (C-14), −4.2 × 2 (C-16, 17).

#### 3.5.2. Desilication of Compound **2a**

To a solution of compound **2a** (6.8 mg, 0.0188 mmol) in dry THF (0.1 mL), TBAF (1 M in THF, 19 μL, 0.0197 mmol, 1.05 equiv) was added at 0 °C. The resulting mixture was stirred at 0 °C for 5 min and then quenched by adding 1.0 mL of the saturated ammonium chloride aqueous solution. The resulting mixture was then extracted by EtOAc (5.0 mL) three times and the combined organic extracts were dried over anhydrous Na_2_SO_4_ and were then concentrated. The residue was purified by using flash column chromatography on the silica gel (200–300 mush, 1.0 × 3.0 cm, petroleum ether/EtOAc 4:1), which yielded compound **2** (4.5 mg, 96.6% yield) as a white solid. Compound **2**: UV (MeOH) *λ*_max_ (log *ε*): 206 (3.81) nm, 218 (3.52) nm, 250 (2.78) nm, 330 (3.29) nm; IR (KBr) *ν*_max_ 3548, 3466, 3169, 2942, 1668, 1602, 1460, 1404, 1229, 1094, 1024, 925, 856; ^1^H-NMR (600 MHz, CDCl_3_): *δ*_H_ 7.17 (1H, s, H-5), 6.16 (1H, s, H-3), 4.08 (3H, s, Me-15), 3.25 (1H, m, H-11), 2.31 (3H, s, Me-14), 1.30 (3H, d, *J* = 6.8 Hz, Me-12), 1.30 (3H, d, *J* = 6.8 Hz, Me-13); ^13^C-NMR (150 MHz, CDCl_3_): *δ*_C_ 163.0 (C-4), 161.5 (C-2), 150.0 (C-7), 145.5 (C-10), 133.6 (C-8), 121.1 (C-6), 120.0 (C-5), 112.2 (C-9), 107.8 (C-3), 61.9 (C-15), 28.7 (C-11), 22.1 (C-12), 22.1 (C-13), 15.9 (C-14), HR-ESI-MS: *m*/*z* 247.0975[M − H]^−^, calcd. 247.0976 for C_14_H_15_O_4_.

#### 3.5.3. Desilication of Compound **3a**

To a solution of compound **3a** (4.2 mg, 0.0116 mmol) in dry THF (0.1 mL), TBAF (1 M in THF, 12 μL, 0.0121 mmol, 1.05 equiv) was added at 0 °C. The resulting mixture was stirred at 0 °C for 5 min and then quenched by adding 1.0 mL of saturated ammonium chloride aqueous solution. The resulting mixture was then extracted by EtOAc (5.0 mL) three times and the combined organic extracts were dried over anhydrous Na_2_SO_4_ and concentrated. The residue was purified by flash column chromatography on the silica gel (200–300 mush, 1.0 × 3.0 cm, petroleum ether/EtOAc 4:1), which yielded compound **3** (2.8 mg, 97.4% yield) as a white solid. Compound **3**: ^1^H-NMR (600 MHz, CDCl_3_): *δ*_H_ 7.01 (1H, s, H-5), 6.22 (1H, s, H-3), 3.95 (3H, s, Me-15), 3.25 (1H, m, H-11), 2.32 (3H, s, Me-14), 1.31 (6H, d, *J* = 6.8 Hz, Me-12, 13); ^13^C-NMR (150 MHz, CDCl_3_): *δ*_C_ 163.0 (C-4), 161.1 (C-2), 147.8 (C-7), 141.3 (C-10), 136.7 (C-8), 127.5 (C-6), 115.5 (C-5), 114.5 (C-9), 109.2 (C-3), 60.5 (C-15), 28.7 (C-11), 21.9 (C-12 & C-13), and 16.3 (C-14).

### 3.6. MTT and CCK-8 Assay

Human HepG2, HeLa, U251, HOS, MG63, U2OS, MB231, and SKBR-3 cells were obtained from the Cell Library of Committee on Type Culture Collection of Chinese Academy of Sciences (Shanghai, China). Cells were cultured at 37 °C, 5% CO_2_ in the Dulbecco’s Modified Eagle’s medium (DMEM), Minimum Eagle’s medium (MEM), or the Roswell Park Memorial Institute (RPMI) medium containing 10% FBS, 100 U/mL penicillin, and 100 U/mL streptomycin.

Compounds **1**–**8** were dissolved in DMSO and diluted with DMEM medium (containing 1% FBS and 100 U/mL penicillin/streptomycin) for certain concentrations (1.5625 μM, 3.125 μM, 6.25 μM, 12.5 μM, 25 μM, 50 μM, and 100 μM). The concentration of DMSO in the final solutions was no more than 0.1%. Human HepG2, HeLa, U251, HOS, MG63, U2OS, MB231, and SKBR-3 cells were seeded in 96-well micro-titer plates (100 μL, 1 × 10^4^ cells/well). When the cells grew to certain concentrations (70%–80% of the well), the medium was removed and the diluted compounds (200 μL) were added to each well. Blank (only medium) and control (cells with DMEM medium) group were set to calculate the cell viability and Taxol was used as a positive control. After 48 h, the 96-well micro-titer plates were taken out from the incubator and the medium was removed. When using the MTT assay, DMEM medium (200 μL) should be first added into the well and then followed by MTT (20 μL, 5 mg/mL dissolved in PBS). After culturing for 4 h, the 96-well micro-titer plates were taken out from the incubator and the medium was removed and then 150 μL of DMSO was added into the well. The absorbance was measured by a microplate reader (Bio-Rad, America) at 560 nm. When using CCK-8 assay, DMEM medium (100 μL) should be first added into the well and then CCK-8 (Dojindo, Kumamoto, Japan) followed. After culturing for 2.5 h, the 96-well micro-titer plates were taken out from the incubator and the absorbance was measured by a microplate reader at 450 nm. The cell viability = (Lab group − Blank group)/(Control group − Blank group) and the IC_50_ value was calculated by the software GraphPad Prism 7.0 with the cell viability value.

### 3.7. Immunoregulation Activity

THP-1 cells were obtained from the Harbin medical university and was cultured in RPMI medium. The cell was seeded in 6-well plate (2 mL, 2 × 10^6^ cell/well) and starved for 12 h. Lipopolysaccharide (LPS, Sigma, St. Louis, MI, USA) (2 mg/mL in PBS) was then added in the well to stimulate the cell. One hour later, each compound was dissolved in DMSO at a concentration of 20 μM. It was added in the well and cultivated for 24 h. The cell was then collected. Following the manufacturer’s instructions, the Trizol reagent (Invitrogen, Carlsbad, CA, USA) was used to isolate the total RNA of THP-1 cell. The extracted total RNA was dissolved in RNA enzyme-free water and added into a 100 μL reaction mixture for reverse transcription into complementary DNA (cDNA). The extracted RNA solution contained 8 μg, 8 μL of 50 pmol/μL Oligo d(T)18 and the volume was brought up to 46 μL with RNA enzyme-free water, incubated at 70 °C for 5 min, and then 4 °C for 5 min. Afterward, 20 μL of 2.5 μmol/mL dNTP, 20 μL of 5× RT buffer, 8 μL of dTT, 2 μL of RNA inhibitor, and 4 μL of M-MLV were added and the mixture was incubated at 42 °C for 3 h. The 100 μL mixture was stored at −20 °C for qualitative PCR (qPCR). Detected via qPCR, the gene expression levels were identified with a LightCycler^®^ 480 System (Roche, Basel, Switzerland) using the TransStart^®^ Tip Green qPCR SuperMix (TRANSGEN BIOTECH, Beijing, China). The cDNA was added into a 20 μL reaction mixture: 10 μL of 2× TransStart^®^ Tip Green qPCR SuperMix, 2 μL of cDNA Template (100–200 pg), 0.8 μL of primer mixture (10 μM), 0.4 μL of Passive Reference Dye (50×), and 6.8 μL of ddH*_2_*O. The PCR procedure includes a temperature of 94 °C and a time period of 30 s followed by 40 cycles at 94 °C for 5 s, 57 °C for 15 s, and 72 °C for 10 s. The β-actin gene was used a control to quantify other genes. The results were calculated using 2^−ΔΔ*C*t^ where ΔΔ*C*t = (*C*t Target − *C*t β-actin)Lab − (*C*t Target − *C*t β-actin) Control. Primers of the target gene TNF-α, IL-1β, and β-actin were designed by Primer 5.0 software following the published gene sequence in GenBank: TNF-α Forward: CAGCAAGGGACAGCAGAGG, Reverse: AGTATGTGAGAGGAAGAGAACC; IL-1β Forward: TGATGGCTTATTACAGTGGCAATG, Reverse: TGATGGCTTATTACAGTGGCAATG; β-actin Forward: ATCGGCAATGAGCGGTTCC, Reverse: ATCGGCAATGAGCGGTTCC.

## 4. Conclusions

In this work, two new phenolic derivatives dryofragone (**1**) and dryofracoumarin B (**2**) were isolated from *Dryopteris fragrans* by cytotoxicity-guided tracking. Two coumarin isomers dryofracoumarin B (**2**) and dryofracoumarin A (**3**) were separated by a silicon etherification-desilication procedure. Compounds (**4**) and (**6**) were first reported in this plant. The cytotoxicity and immuno-regulation activity were examined among the eight compounds and the relationship between cytotoxicity and immuno-regulation activity revealed that compounds may activate the LPS signaling to regulate the growth of tumor cells through immuno-regulation. This relation needs further study.

## Figures and Tables

**Table 1:** NMR data for Compound **1** (TMS as the internal standard, *δ* in ppm, *J* in Hz) ^a^.

No.	*δ*_H_	*δ*_C_	No.	*δ*_H_	*δ*_C_
1		196.32 (C)	8a	2.99 (1H, m)	41.04 (CH_2_)
2		75.37 (C)	8b	2.92 (1H, m)	
3		176.04 (C)	9	1.69 (2H, m)	18.71 (CH_2_)
4	5.37 (1H, s)	94.49 (CH)	10	1.01 (3H, t, *J* = 7.4)	14.07 (CH_3_)
5		189.34 (C)	11	1.54 (3H, s)	30.20 (CH_3_)
6		104.49 (C)	12	3.91 (3H, s)	57.35 (CH_3_)
7		203.65 (C)			

^a^^1^H-NMR and ^13^C-NMR data were recorded in CDCl_3_ at 600 MHz and 150 MHz, respectively.

**Table 2:** NMR data for compound **2** and **3** (TMS as the internal standard, *δ* in ppm, *J* in Hz) ^a^.

No.	2		3	
	*δ*_H_	*δ*_C_	*δ*_H_	*δ*_C_
1				
2		161.5 (C)		161.1 (C)
3	6.16 (1H, s)	107.8 (CH)	6.22 (1H, s)	109.2 (CH)
4		163.0 (C)		163.0 (C)
5	7.17 (1H, s)	120.0 (CH)	7.01 (1H, s)	115.5 (CH)
6		121.1 (C)		127.5 (C)
7		150.0 (C)		147.8 (C)
8		133.6 (C)		136.7 (C)
9		112.2 (C)		114.5 (C)
10		145.5 (C)		141.3 (C)
11	3.25 (1H, m)	28.7 (CH)	3.25 (1H, m)	28.7 (CH)
12	1.30 (3H, d, *J* = 6.8)	22.1 (CH_3_)	1.31 (3H, d, *J* = 6.8)	21.9 (CH_3_)
13	1.30 (3H, d, *J* = 6.8)	22.1 (CH_3_)	1.31 (3H, d, *J* = 6.8)	21.9 (CH_3_)
14	2.31 (3H, s)	15.9 (CH_3_)	2.32 (3H, s)	16.3 (CH_3_)
15	4.08 (3H, s)	61.9 (OCH_3_)	3.95 (3H, s)	60.5 (OCH_3_)

^a^^1^H-NMR and ^13^C-NMR data were recorded in CDCl_3_ at 600 MHz and 150 MHz, respectively.

**Table 3:** IC_50_ values (μM) of cytotoxicity for eight compounds against nine human cancer cell lines.

Compound	HepG2	A549	HeLa	U251	HOS	MG63	U2OS	MB231	SKBR-3
**1**	-	45.86 ± 1.64	25.37 ± 2.62	46.13 ± 1.90	-	-	-	-	-
**2**	45.52 ± 3.21	47.70 ± 2.43	15.12 ± 4.01	46.14 ± 2.40	-	-	-	25.59 ± 2.30	-
**3**	48.39 ± 2.15	38.01 ± 3.56	-	-	-	-	-	38.09 ± 2.40	-
**4**	38.13 ± 1.03	37.41 ± 1.24	1.24 ± 0.08	-	-	-	47.56 ± 2.23	41.95 ± 2.35	-
**5**	-	49.74 ± 3.35	-	-	-	-	-	-	-
**6**	-	47.42 ± 2.25	-	-	-		-	-	-
**7**	-	40.03 ± 0.98	-	-	-	-	-	-	
**8**	18.02 ± 0.89	-	17.76 ± 3.43	41.21 ± 1.35	43.67 ± 2.52	22.76 ± 2.65	36.36 ± 1.32	39.61 ± 1.50	33.40 ± 1.50
Taxol ^a^	5.32 ± 0.12	3.46 ± 0.23	0.17 ± 0.02	5.02 ± 0.21	3.71 ± 0.33	5.86 ± 0.24	1.01 ± 0.03	6.23 ± 0.36	3.12 ± 0.25

Note: IC_50_ values represented the means ± SD of six independent experiments and “-” means the IC_50_ value is above 50 μM. ^a^ Taxol was used as a positive control.

## Supplementary Materials

The following are available online. Supplementary materials included Figures S1 and S2: Two round screening of cytotoxicity with MTT or CCK-8 assay, Figures S3–S27: Spectrum and Spectroscopy data of compounds **1**–**3**, **2a** and **3a**, and NMR spectral data of compounds **4**–**8**.
