# Supplementary material for: Cytotoxicity-Guided Isolation of Two New Phenolic Derivatives from Dryopteris fragrans (L.) Schott
Source: Molecules. 2018 Jul 6;23(7):1652. doi: 10.3390/molecules23071652 (PMC6100289; doi:10.3390/molecules23071652)
Supplement: Supplementary file 1 [file molecules-23-01652-s001.pdf]

Supplementary material

Cytotoxity-guided isolation of two new phenolic derivatives from *Dryopteris fragrans* (L.) Schott

Tong Zhang 1, Li Wang 2, De-Hua Duan 1, Yi-Hao Zhang 1, Sheng-Xiong Huang 2,* and Ying Chang 1,*

[1] College of Life Science, Northeast Agricultural University, Harbin 150030, China; shmzhyzt@163.com (T.Z.); myrddh@163.com(D.-H.D.); yhzneau@163.com(Y.-H.Z.)

[2] State Key Laboratory of Phytochemistry and Plant Resources in West China, Kunming Institute of Botany, Chinese Academy of Sciences, Kunming 650201, China; jasminewangli@126.com (L.W.)

* Correspondence: sxhuang@mail.kib.ac.cn (S.-X.H.); changying@neau.edu.cn (C.Y.); Tel.: +86-871-6521-5112 (S.-X.H.); +86-451-5519-0410 (C.Y.)

Contents of Supporting Information

No.	Contents	Page
1	Figure S1. The first round screening of cytotoxic sites of crude extract from *Dryopteris fragrans*.	3
2.	Figure S2. The second round screening of cytotoxic sites of fractions from *Dryopteris fragrans*.	3-4
3.	Figure S3. ^{1}H NMR spectrum of compound **1** recorded in $CDCl_3$ at 600 MHz.	5
4.	Figure S4. ^{13}C NMR spectrum of compound **1** recorded in $CDCl_3$ at 150 MHz.	5
5.	Figure S5. HSQC spectrum of compound **1** recorded in $CDCl_3$ at 600 MHz.	6
6.	Figure S6. HMBC spectrum of compound **1** recorded in $CDCl_3$ at 600 MHz.	6
7	Figure S7. UV spectrum of compound **1**.	7
8	Figure S8. HR-ESI-MS spectrum of compound **1**.	8
9	Figure S9. CD spectrum of compound **1**.	9
10	Figure S10. IR spectrum of compound **1**.	9
11	Figure S11. ^{1}H NMR spectrum of the mixture (**2** and **3**) recorded in $CDCl_3$ at 400 MHz.	10
12	Figure S12. ^{13}C NMR spectrum of the mixture (**2** and **3**) recorded in $CDCl_3$ at 100 MHz.	10
13	Figure S13. ESI-MS spectrum of the mixture (**2** and **3**).	11
14	Figure S14. ^{1}H NMR spectrum of compound **2a** recorded in $CDCl_3$ at 400 MHz.	11
15	Figure S15. ^{13}C NMR spectrum of compound **2a** recorded in $CDCl_3$ at 100 MHz.	12
16	Figure S16. ^{1}H NMR spectrum of compound **2** recorded in $CDCl_3$ at 600 MHz.	12
17	Figure S17. ^{13}C NMR spectrum of compound **2** recorded in $CDCl_3$ at 150 MHz.	13
18	Figure S18. HSQC spectrum of compound **2** recorded in $CDCl_3$ at 600 MHz.	13
19	Figure S19. HMBC spectrum of compound **2** recorded in $CDCl_3$ at 600 MHz.	14
20	Figure S20. ^{1}H-^{1}H COSY spectrum of compound **2** recorded in $CDCl_3$ at 600 MHz.	14
21	Figure S21. UV spectrum of compound **2**.	15
22	Figure S22. HR-ESI-MS spectrum of compound **2**.	16
23	Figure S23. IR spectrum of compound **2**.	17
24	Figure S24. ^{1}H NMR spectrum of compound **3a** recorded in $CDCl_3$ at 400 MHz.	17
25	Figure S25. ^{13}C NMR spectrum of compound **3a** recorded in $CDCl_3$ at 100 MHz.	18
26	Figure S26. ^{1}H NMR spectrum of compound **3** recorded in $CDCl_3$ at 600 MHz.	18
27	Figure S27. ^{13}C NMR spectrum of compound **3** recorded in $CDCl_3$ at 150 MHz.	19
28	^{1}H NMR and ^{13}C NMR spectral data of compounds **4-8**.	19–20

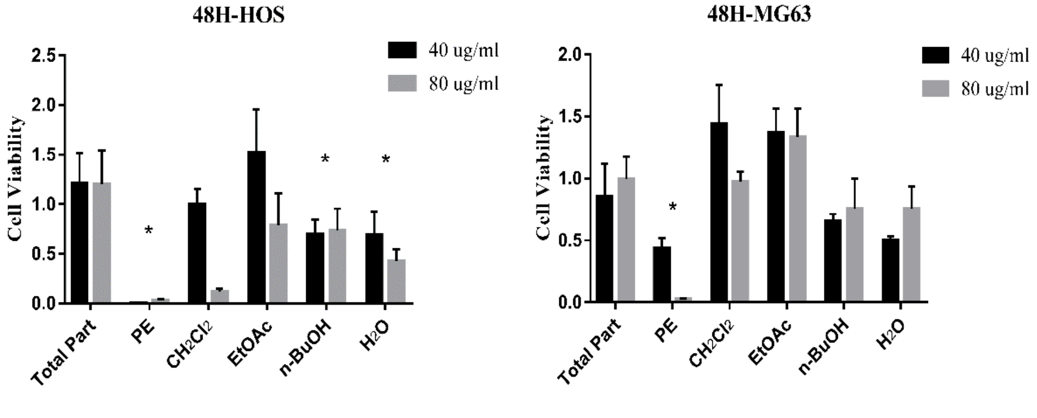

A B

Figure S1. The first round screening of cytotoxic sites of crude extract from *Dryopteris fragrans*. A) against HOS cell line, B) against MG63 cell line. The asterisk indicated that there were significant differences ($p < 0.05$) between other crude extract and the total part. Each value represented the means ±SD of six independent experiments.

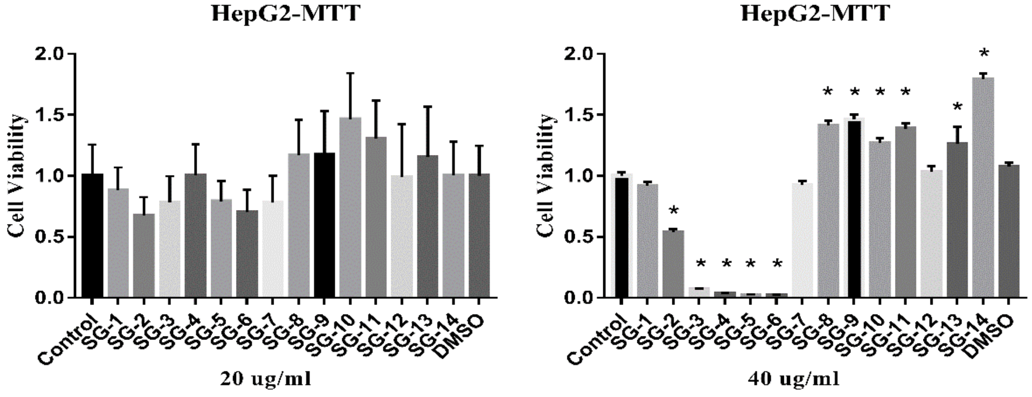

A B

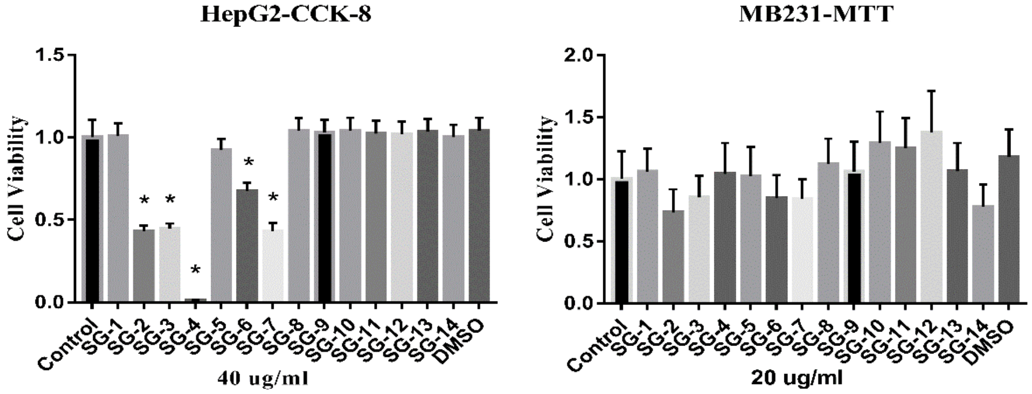

C D

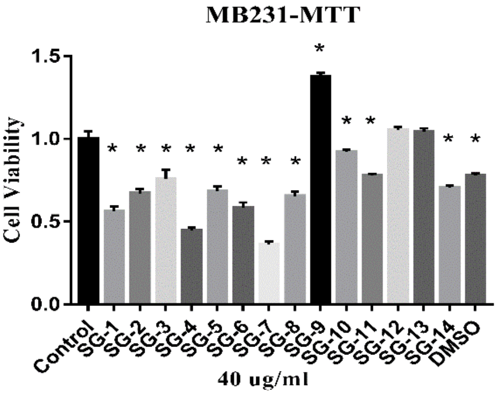

E

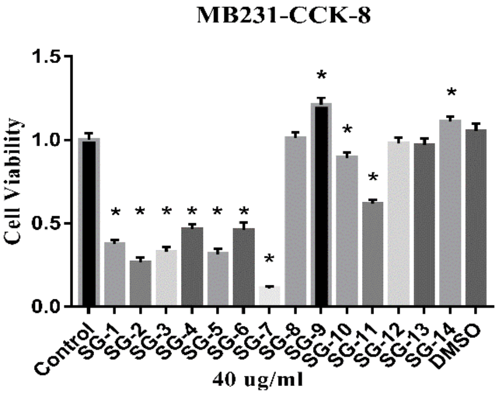

F

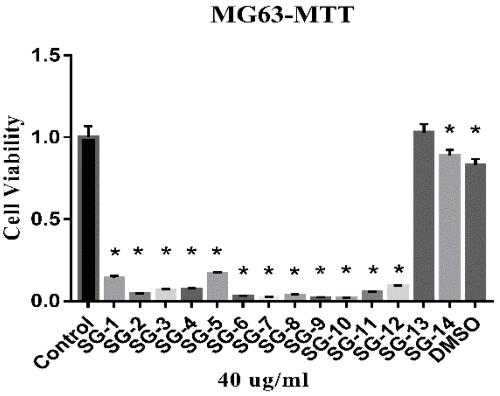

G

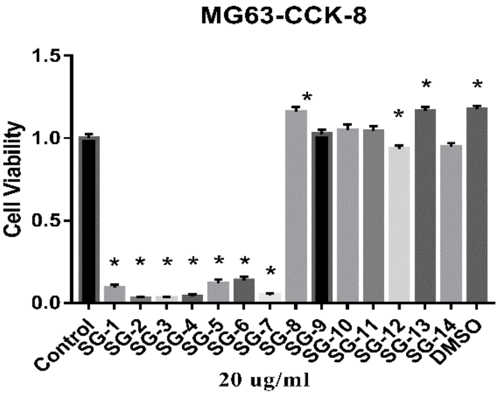

H

Figure S2. The second round screening of cytotoxic sites of fractions from *Dryopteris fragrans*. A) against HepG2 cell line in MTT assay at the concentration of 20 μg/mL; B) against HepG2 cell line in MTT assay at the concentration of 40 μg/mL; C) against HepG2 cell line in CCK-8 assay at the concentration of 40 μg/mL; D) against MB231 cell line in MTT assay at the concentration of 20 μg/mL; E) against MB231 cell line in MTT assay at the concentration of 40 μg/mL; F) against MB231 cell line in CCK-8 assay at the concentration of 40 μg/mL; G) against MG63 cell line in MTT assay at the concentration of 40 μg/mL; H) against MG63 cell line in CCK-8 assay at the concentration of 20 μg/mL. The asterisk indicates that there were significant differences ($p < 0.05$) between other fracions and the control group. Each value represented the means ±SD of six independent experiments.

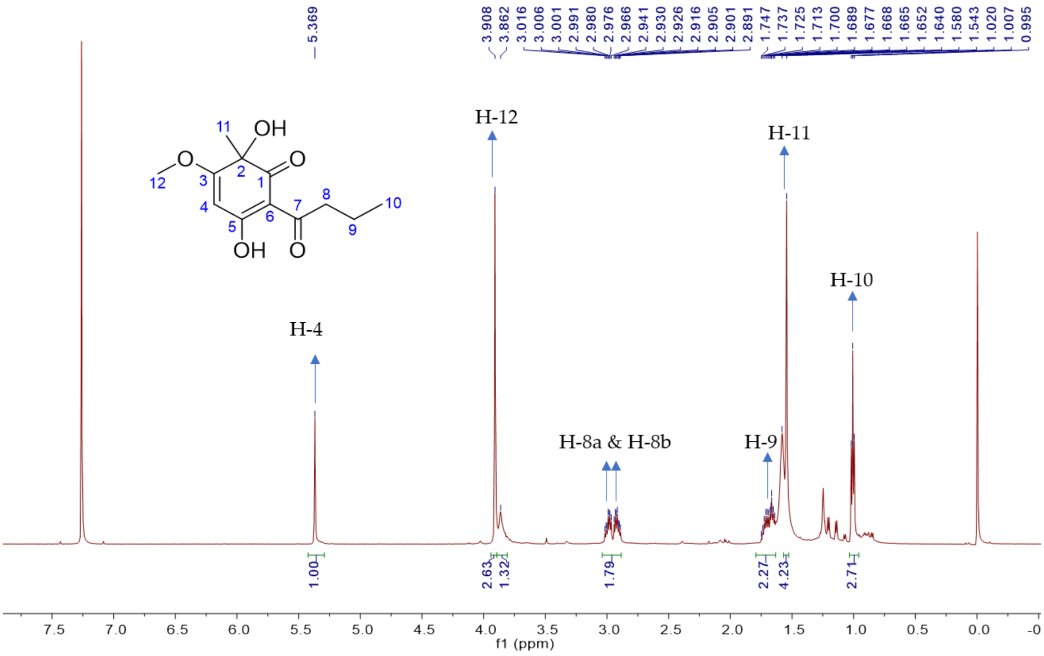

Figure S3. ^{1}H-NMR spectrum of compound **1** recorded in $CDCl_3$ at 600 MHz.

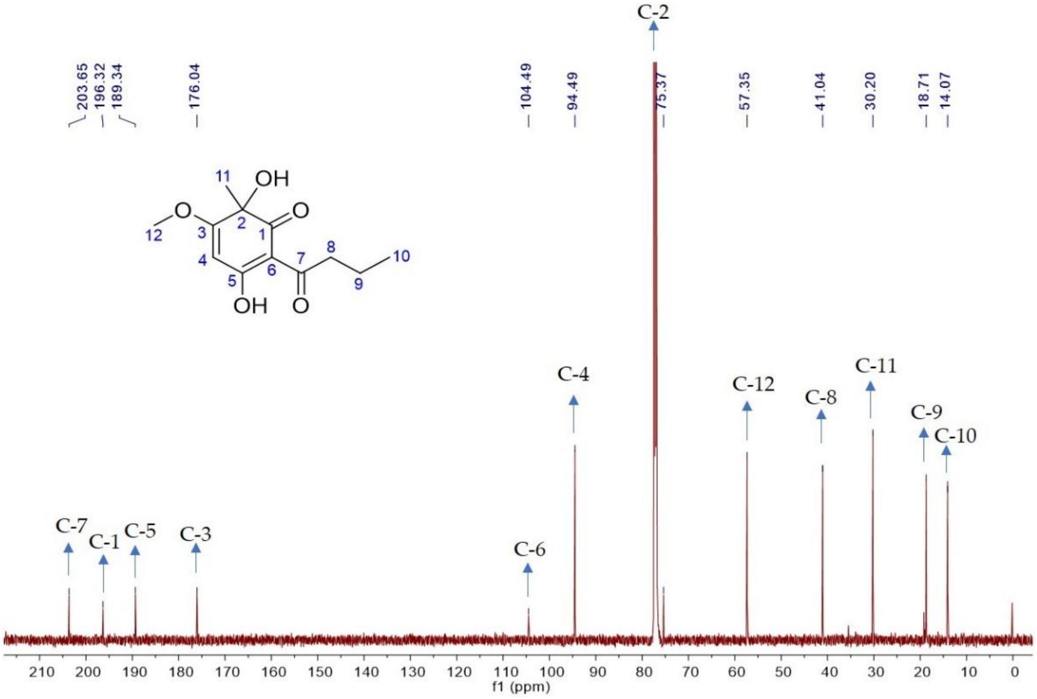

Figure S4. ^{13}C-NMR spectrum of compound **1** recorded in $CDCl_3$ at 150 MHz.

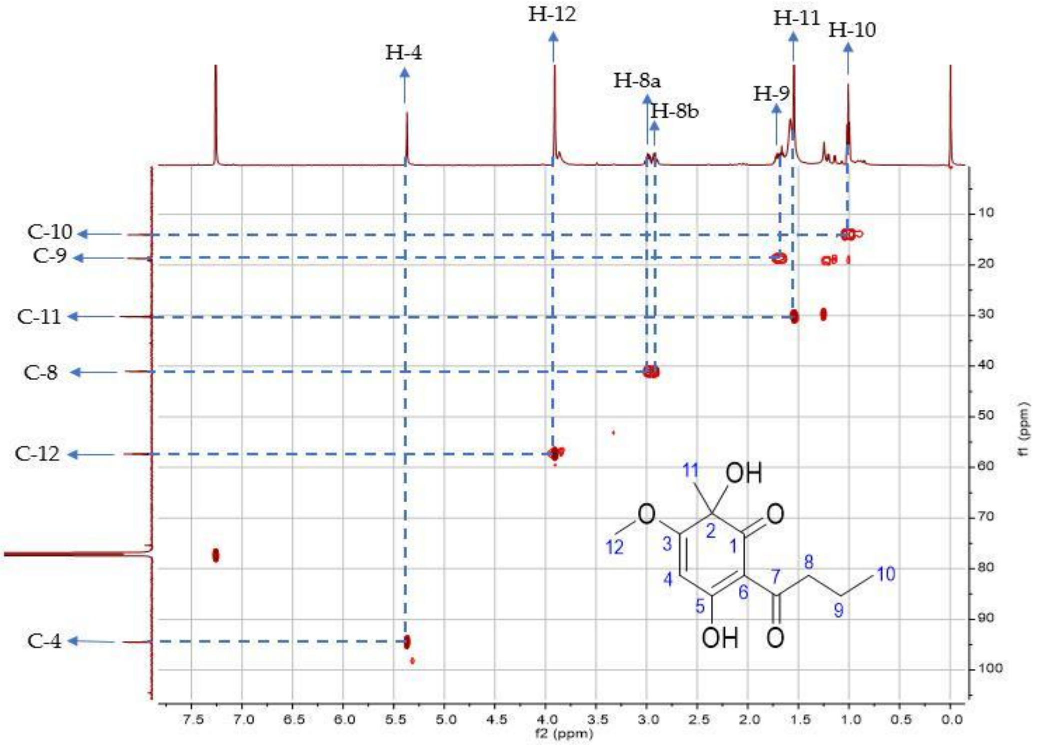

Figure S5. HSQC spectrum of compound **1** recorded in $CDCl_3$ at 600 MHz.

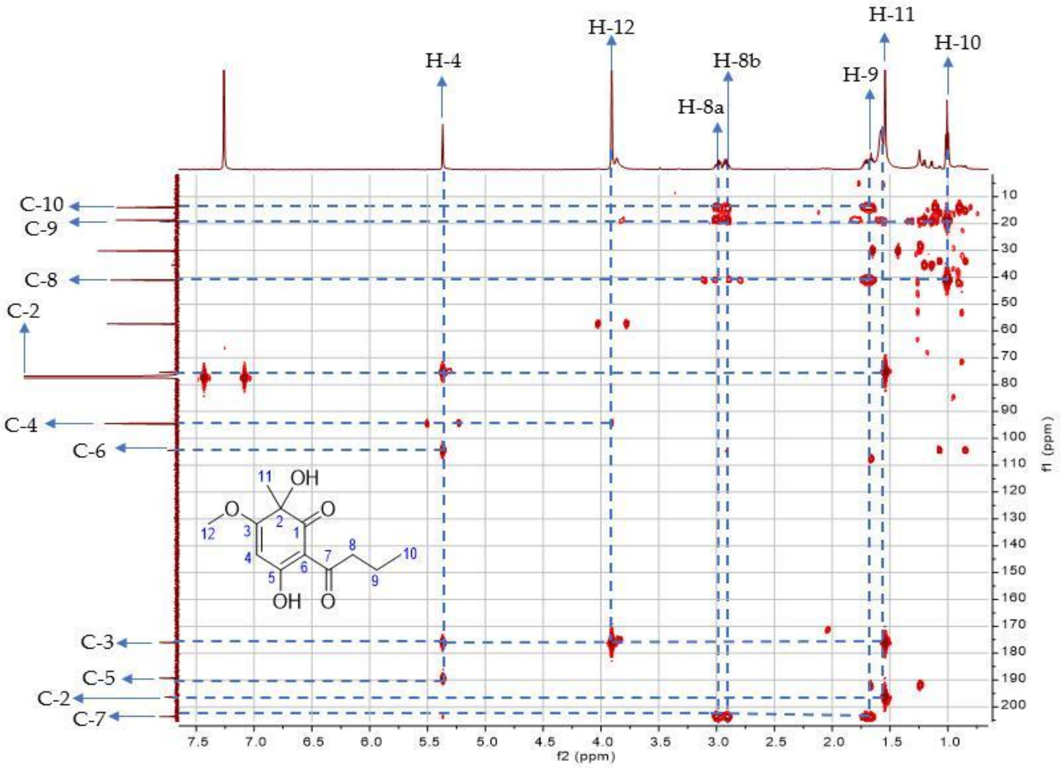

Figure S6. HMBC spectrum of compound **1** recorded in $CDCl_3$ at 600 MHz.

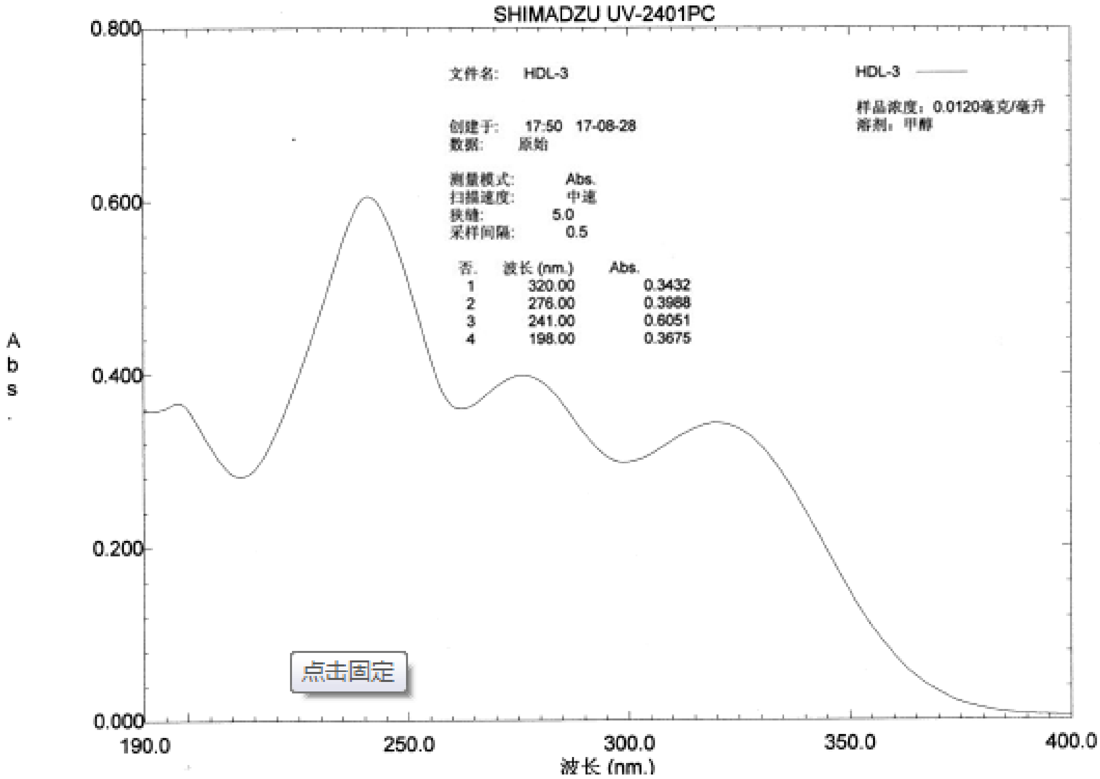

Figure S7. UV spectrum of compound **1**.

Data File: E:\DATA\2017\0822\HDL-3.lcd

Elmt	Val.	Min	Max	Elmt	Val.	Min	Max	Elmt	Val.	Min	Max	Elmt	Val.	Min	Max	Use Adduct
H	1	0	150	O	2	0	50	P	3	0	0	Br	1	0	0	H
B	3	0	0	F	1	0	0	S	2	0	0	I	3	0	5	
C	4	0	100	Na	1	0	0	Cl	1	0	0	Pt	2	0	5	
N	3	0	20	Mg	2	0	0	Fe	2	0	0					

Error Margin (ppm): 10
HC Ratio: unlimited
Max Isotopes: all
MSn Iso RI (%): 75.00
DBE Range: -2.0 - 100.0
Apply N Rule: yes
Isotope RI (%): 1.00
MSn Logic Mode: AND
Electron Ions: both
Use MSn Info: yes
Isotope Res: 10000
Max Results: 10

Event#: 2 MS(E-) Ret. Time : 0.450 Scan# : 92

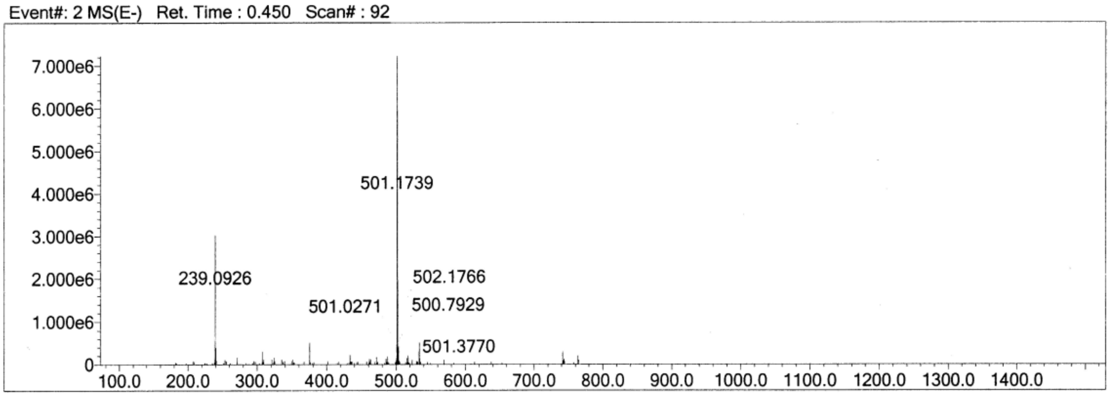

Measured region for 239.0926 m/z

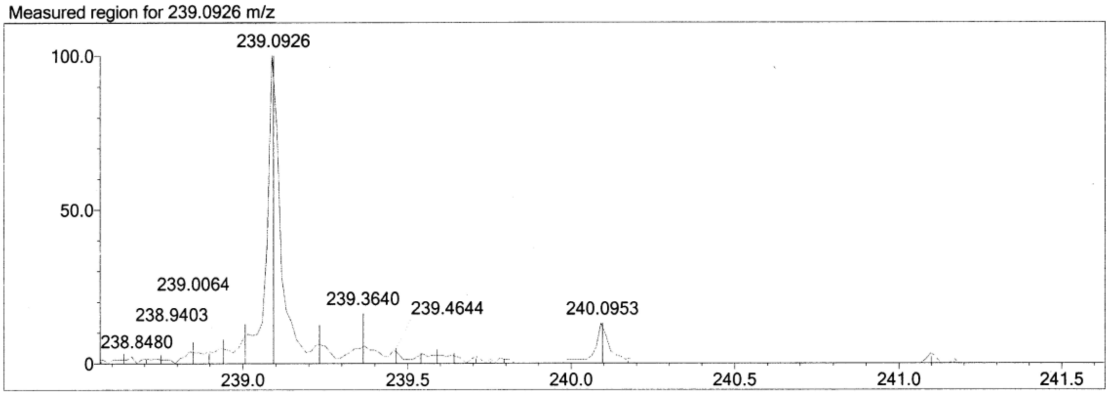

C12 H16 O5 [M-H]- : Predicted region for 239.0925 m/z

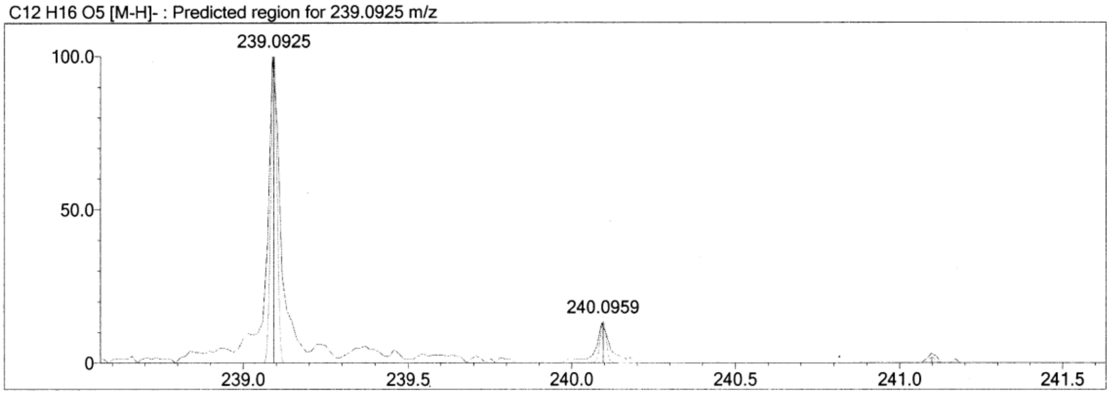

Formula (M)	Ion	Meas. m/z	Pred. m/z	Df. (mDa)	Df. (ppm)	DBE
C12 H16 O5	[M-H]-	239.0926	239.0925	0.1	0.42	5.0

Figure S8. HR-ESI-MS spectrum of compound **1**

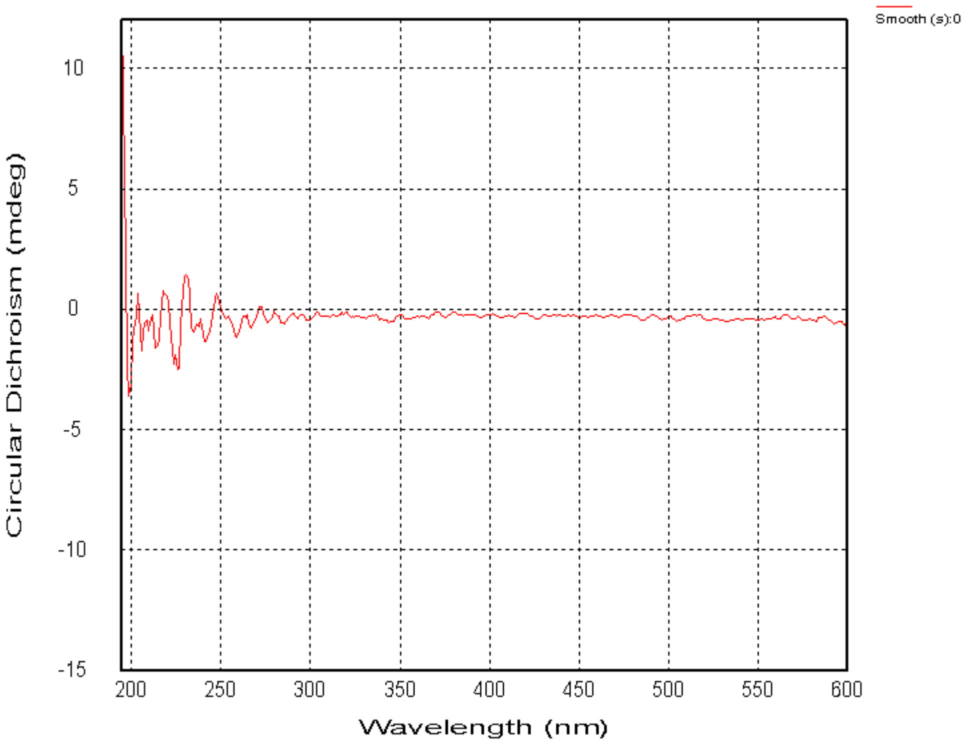

Figure S9. CD spectrum of compound **1**.

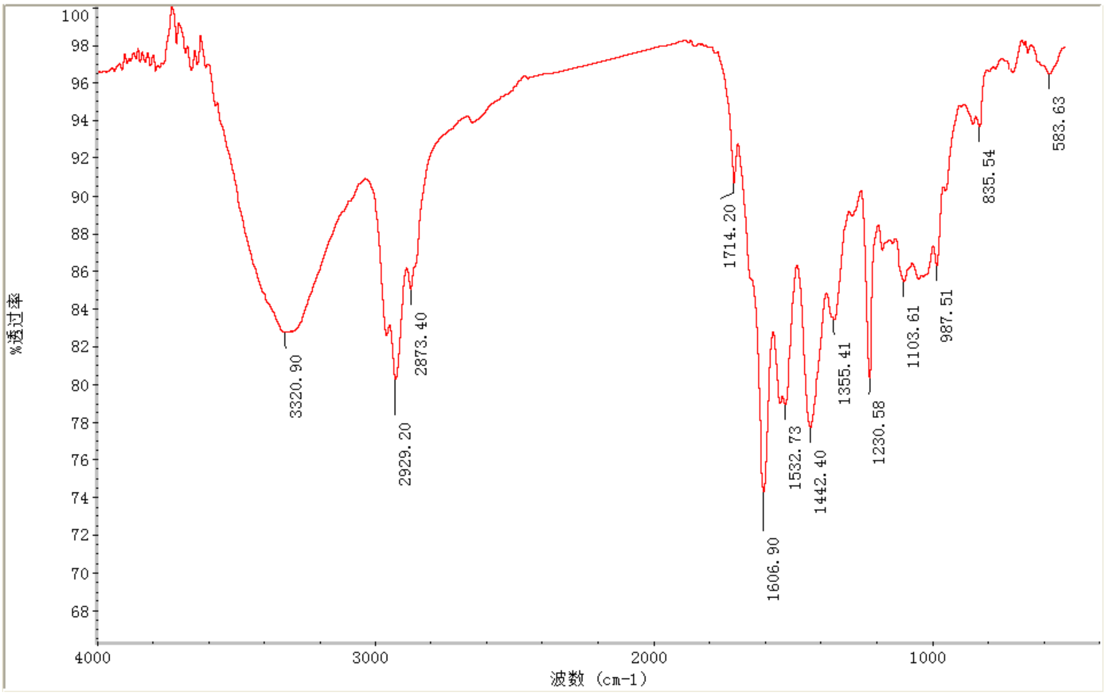

Figure S10. IR spectrum of compound **1**.

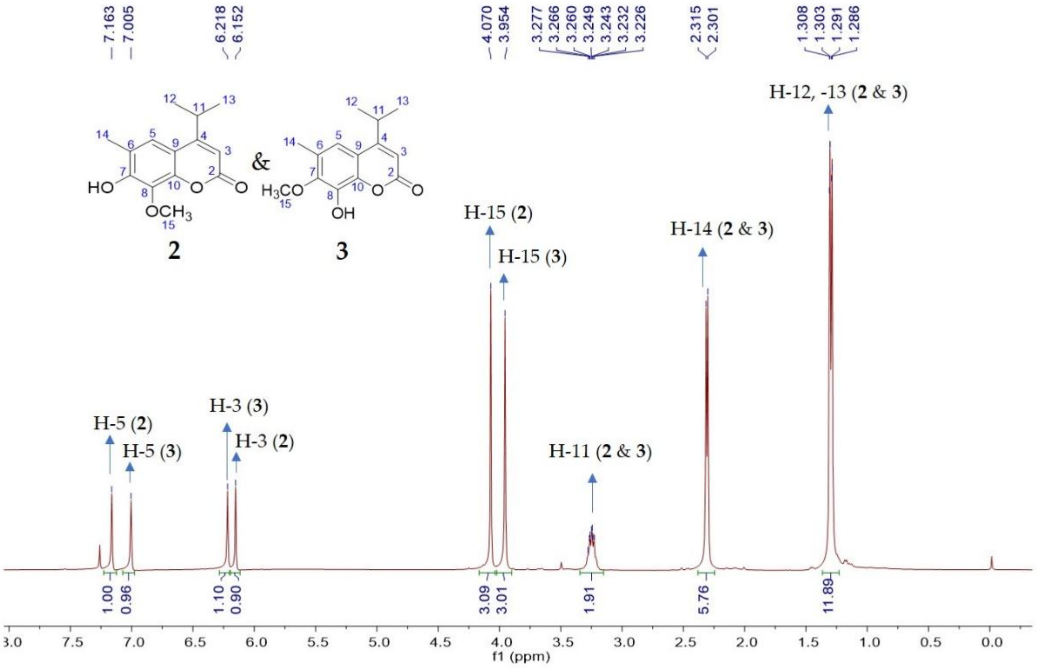

Figure S11. ^{1}H-NMR spectrum of the mixture (**2** and **3**) recorded in $CDCl_3$ at 400 MHz

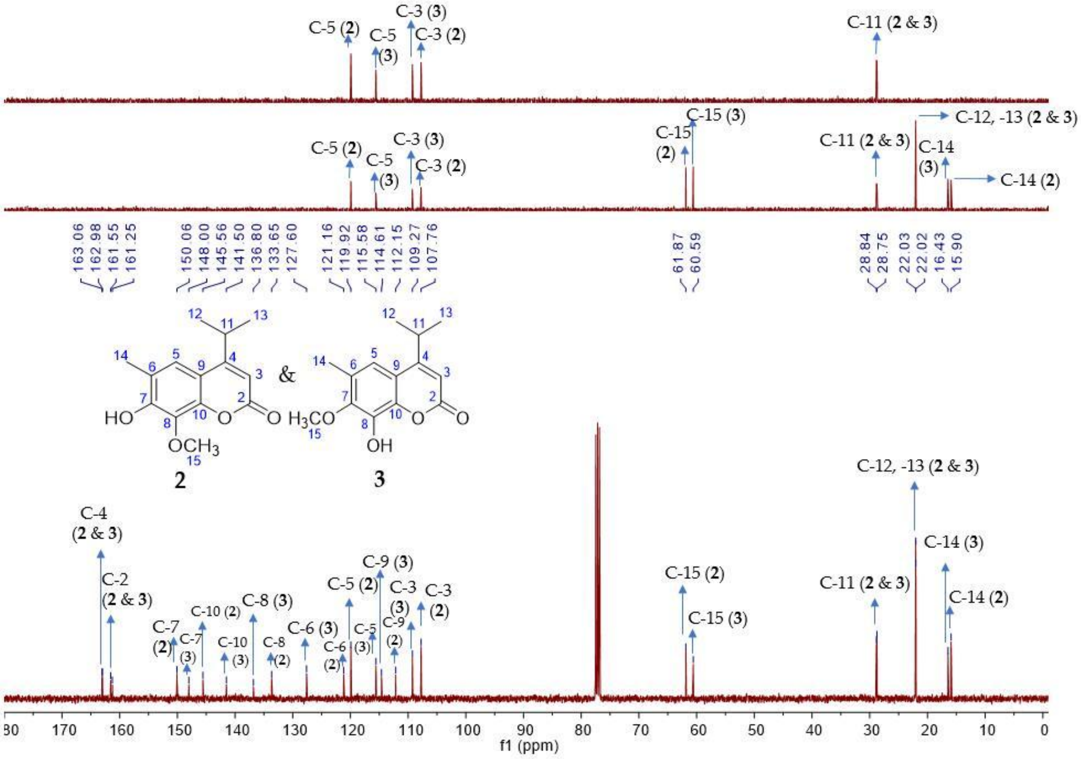

Figure S12. ^{13}C-NMR spectrum of the mixture (**2** and **3**) recorded in $CDCl_3$ at 100 MHz

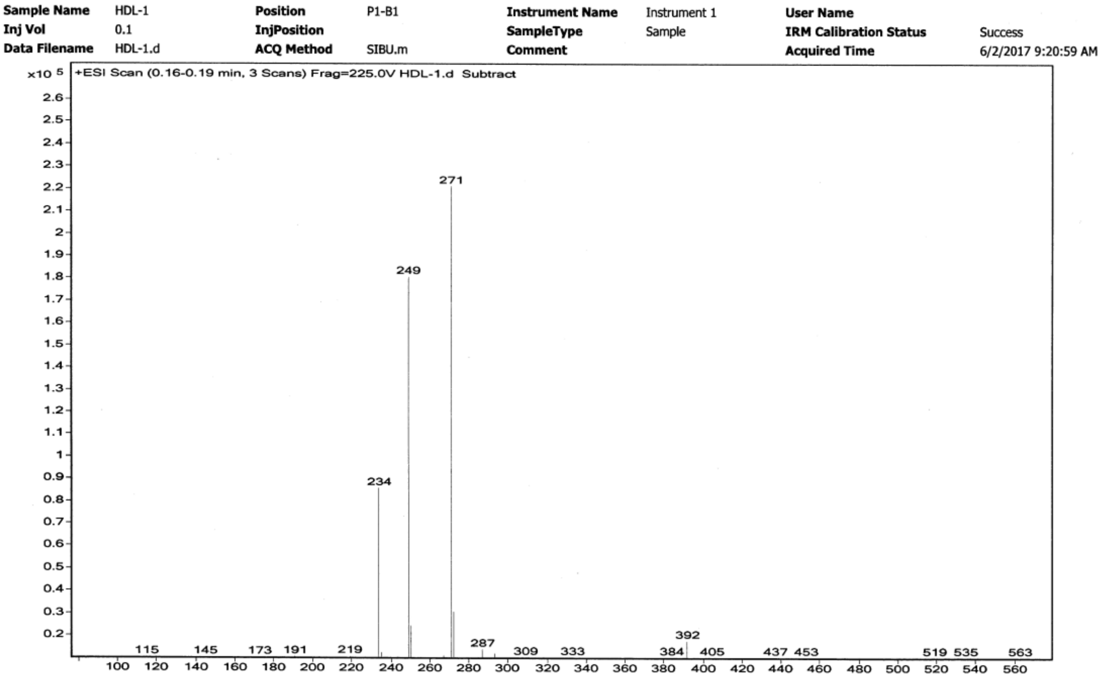

Figure S13. ESI-MS spectrum of the mixture (**2** and **3**).

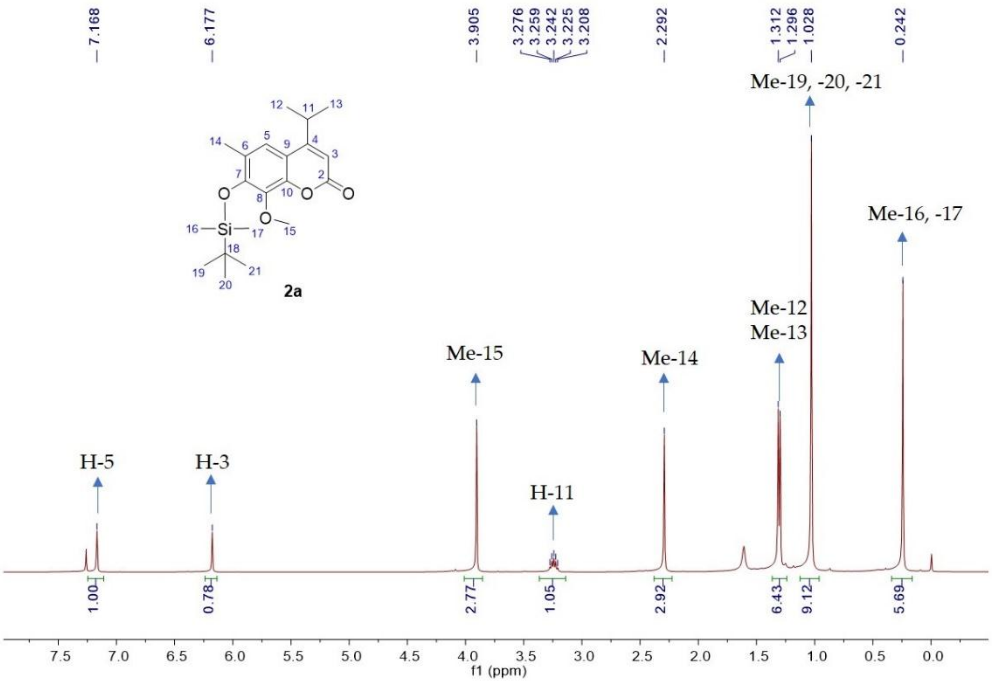

Figure S14. ^{1}H-NMR spectrum of compound **2a** recorded in $CDCl_3$ at 400 MHz.

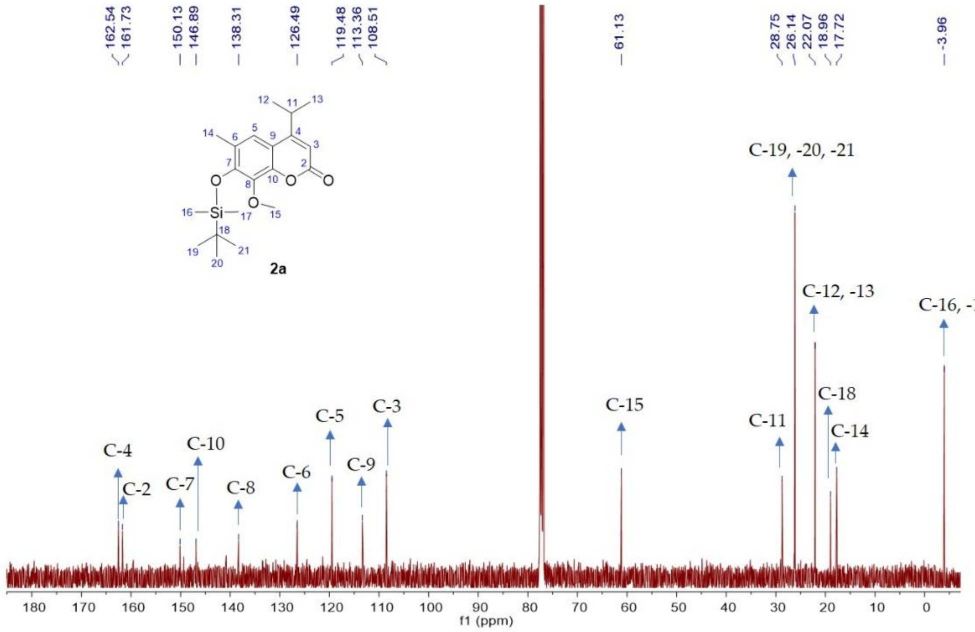

Figure S15. ^{13}C-NMR spectrum of compound **2a** recorded in $CDCl_3$ at 100 MHz.

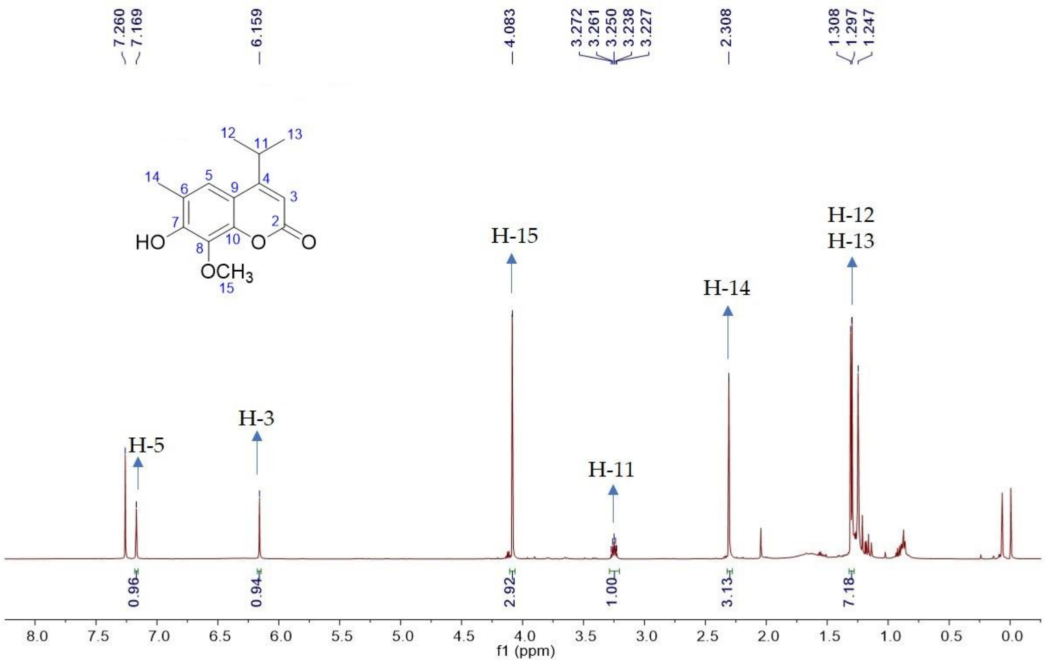

Figure S16. ^{1}H-NMR spectrum of compound **2** recorded in $CDCl_3$ at 600 MHz.

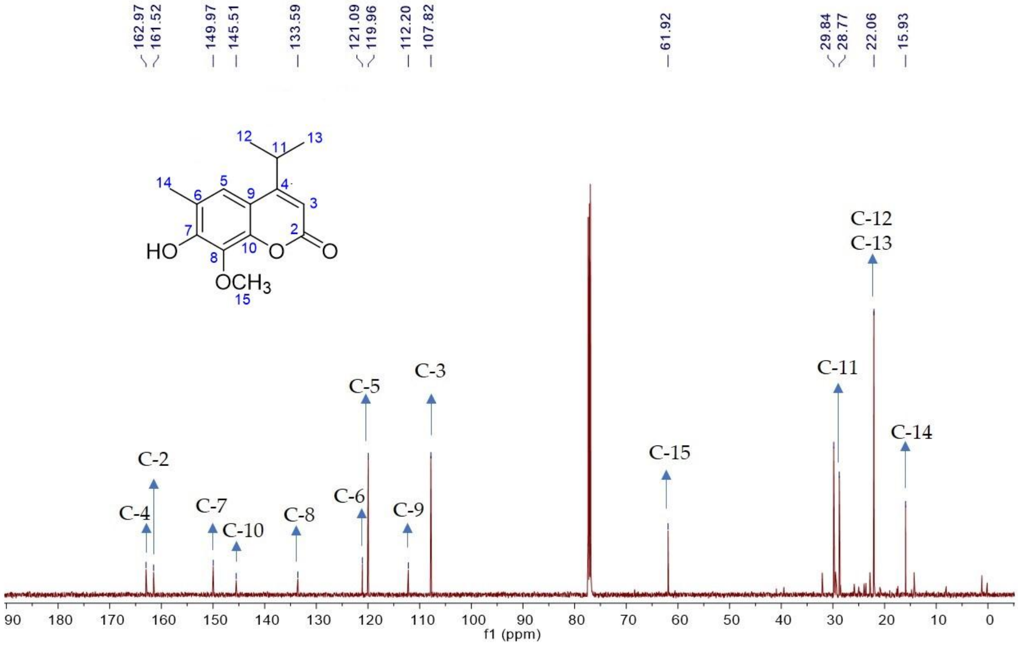

Figure S17. ^{13}C-NMR spectrum of compound **2** recorded in $CDCl_3$ at 150 MHz.

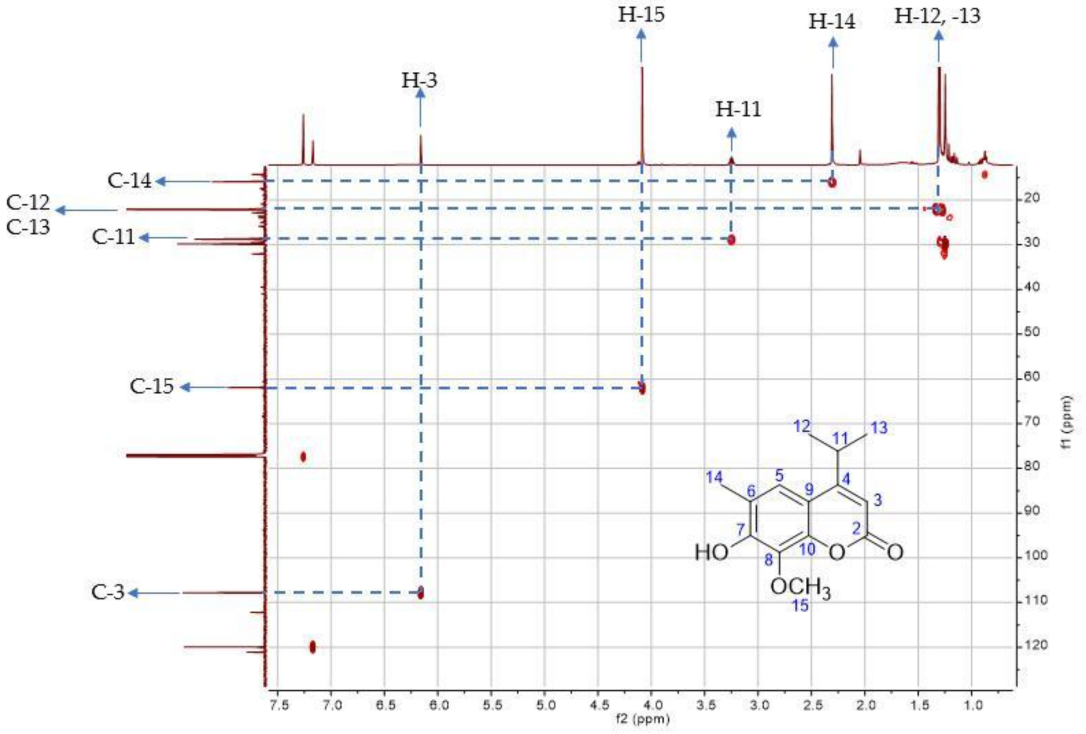

Figure S18. HSQC spectrum of compound **2** recorded in $CDCl_3$ at 600 MHz.

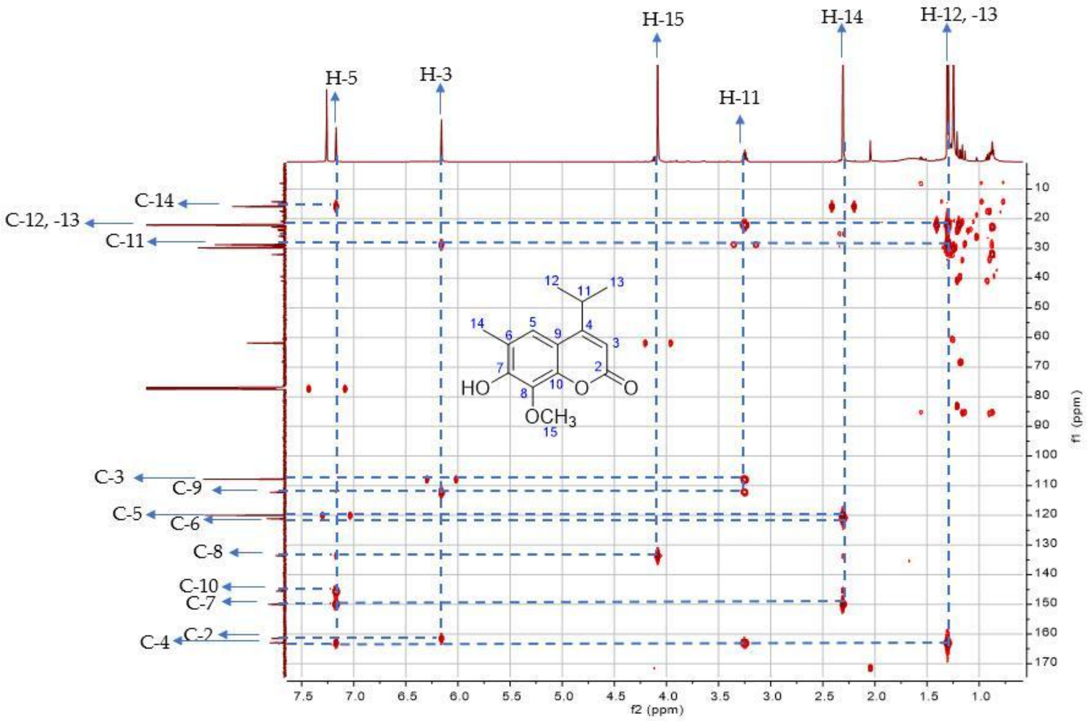

Figure S19. HMBC spectrum of compound **2** recorded in $CDCl_3$ at 600 MHz.(Note: δ_H 1.25 (3H, s), δ_C 29.8 is the impurity signal of "grease")

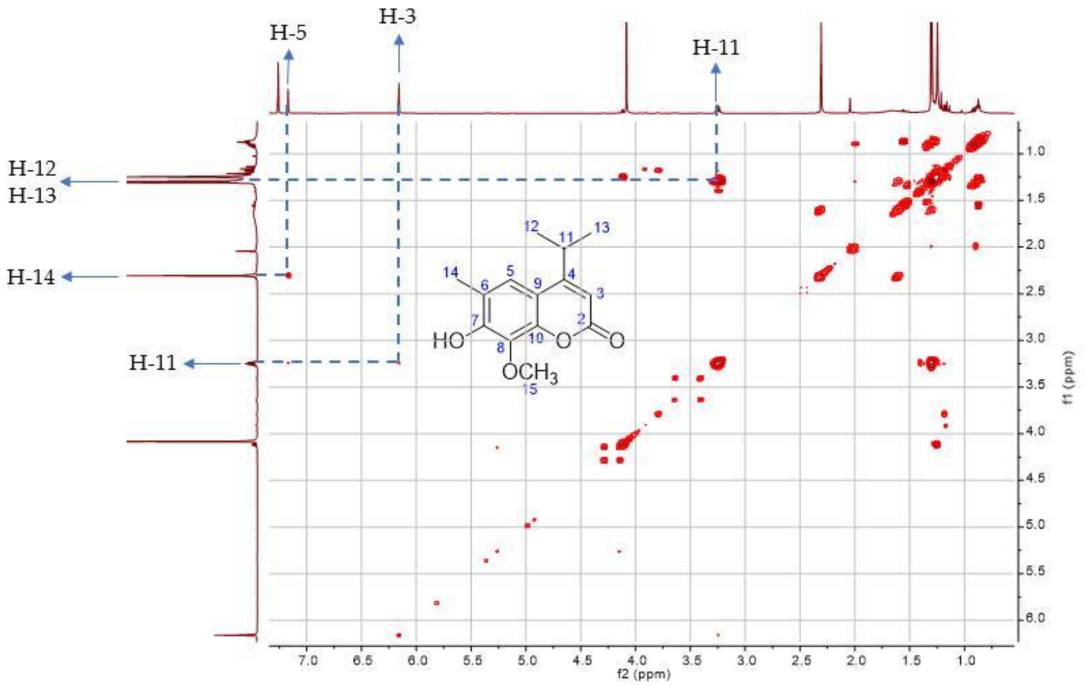

Figure S20. ^1H-^1H COSY spectrum of compound **2** recorded in $CDCl_3$ at 600 MHz.

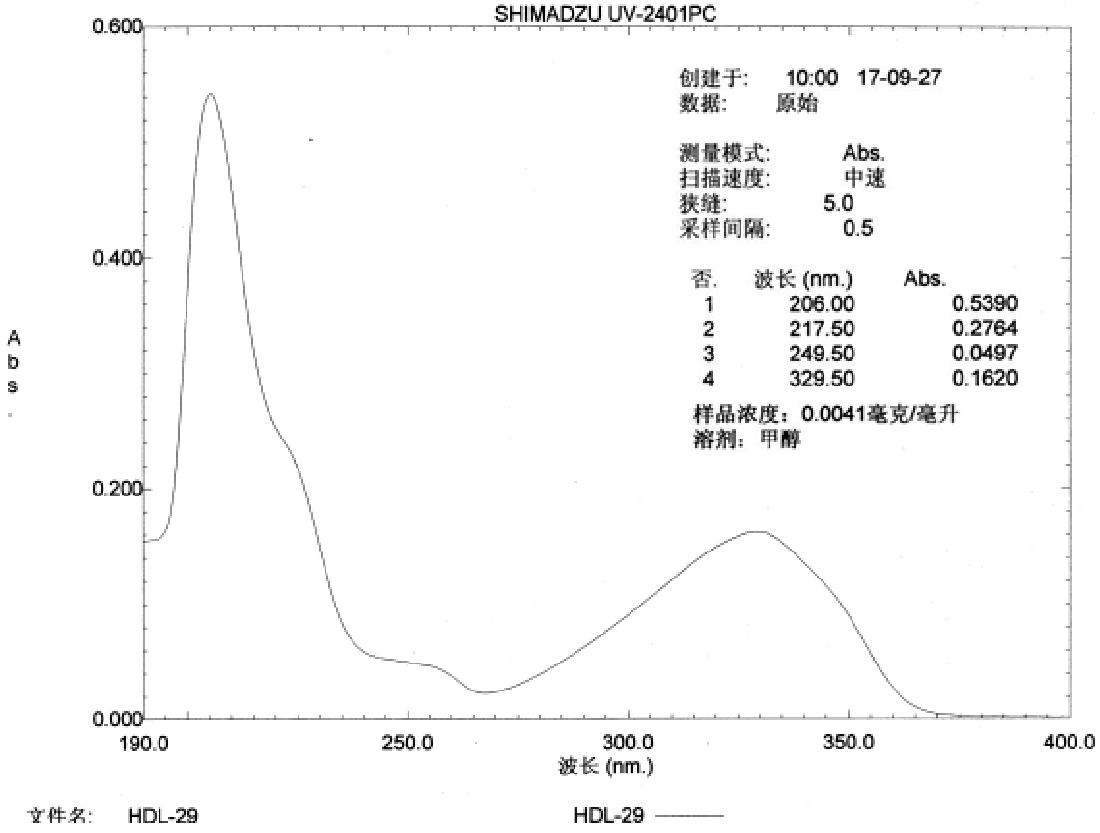

Figure S21. UV spectrum of compound **2**.

Data File: E:\DATA\2017\0926\HDL-29.lcd

Elmt	Val.	Min	Max	Elmt	Val.	Min	Max	Elmt	Val.	Min	Max	Elmt	Val.	Min	Max	Use Adduct
H	1	0	100	O	2	0	50	P	3	0	0	Br	1	0	0	H
B	3	0	0	F	1	0	0	S	2	0	5	I	3	0	0	
C	4	0	100	Na	1	0	0	Cl	1	0	0	Pt	2	0	0	
N	3	0	0	Mg	2	0	0	Fe	2	0	0					

Error Margin (ppm): 10
HC Ratio: unlimited
Max Isotopes: all
MSn Iso RI (%): 75.00

DBE Range: -2.0 - 100.0
Apply N Rule: yes
Isotope RI (%): 1.00
MSn Logic Mode: AND

Electron Ions: both
Use MSn Info: yes
Isotope Res: 10000
Max Results: 10

Event#: 2 MS(E-) Ret. Time : 0.470 Scan# : 96

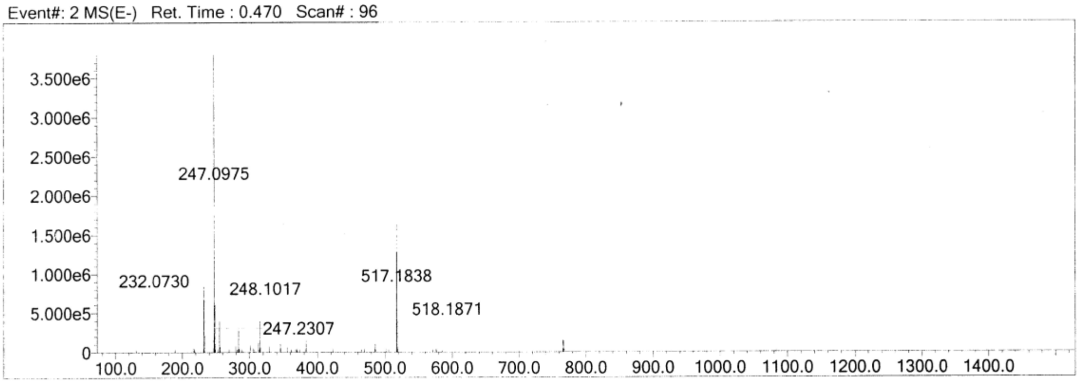

Measured region for 247.0975 m/z

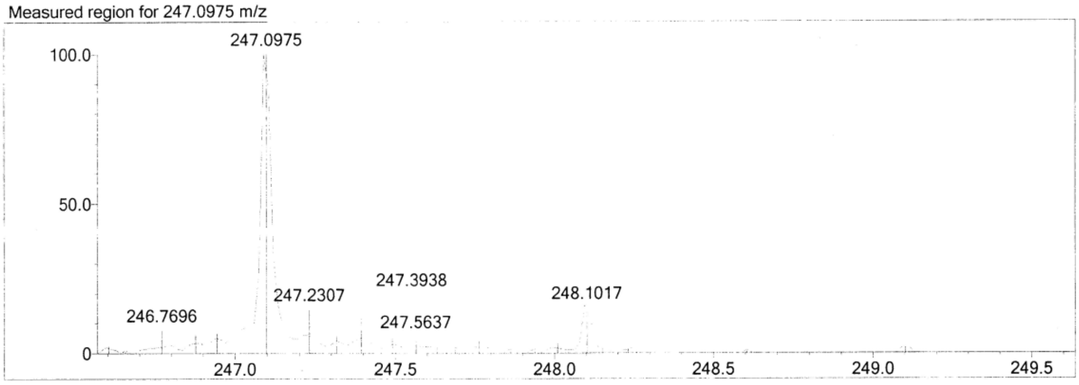

C14 H16 O4 [M-H]- : Predicted region for 247.0976 m/z

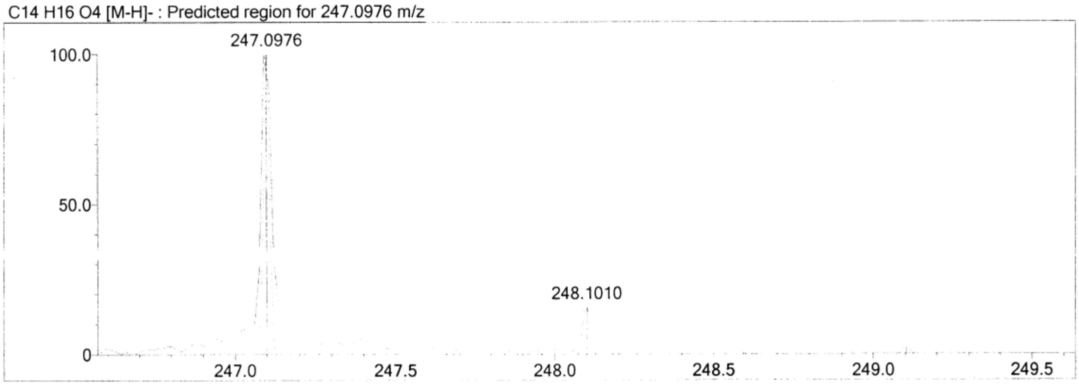

Formula (M)	Ion	Meas. m/z	Pred. m/z	Df. (mDa)	Df. (ppm)	DBE
C14 H16 O4	[M-H]-	247.0975	247.0976	-0.1	-0.40	7.0

Figure S22. HR-ESI-MS spectrum of compound **2**.

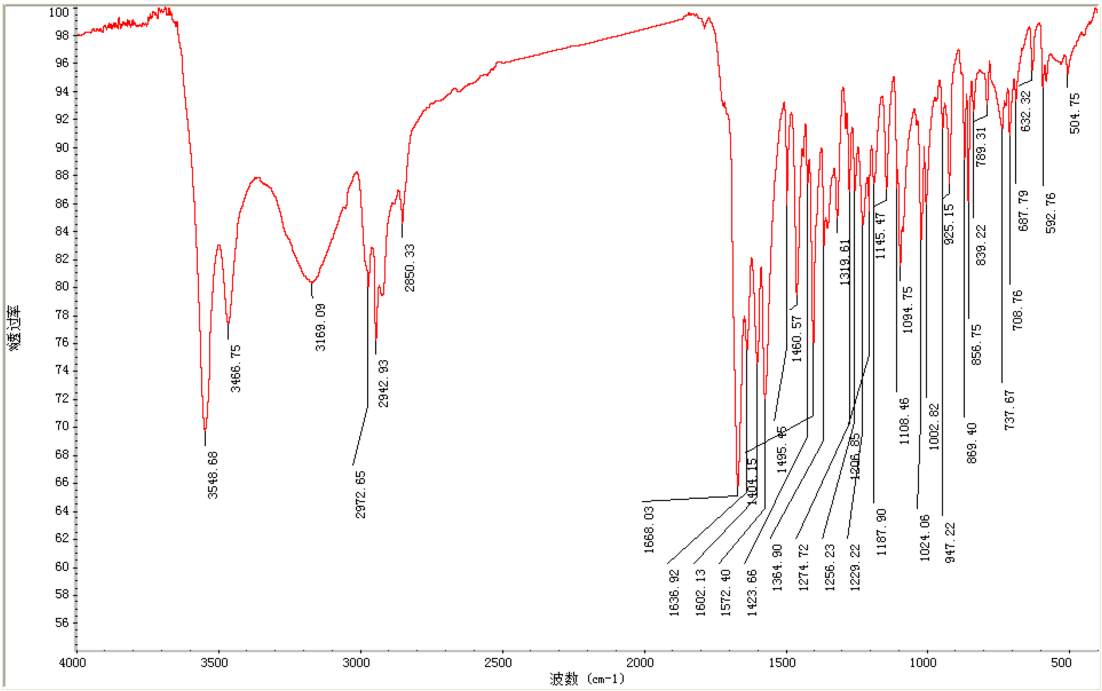

Figure S23. IR spectrum of compound **2**.

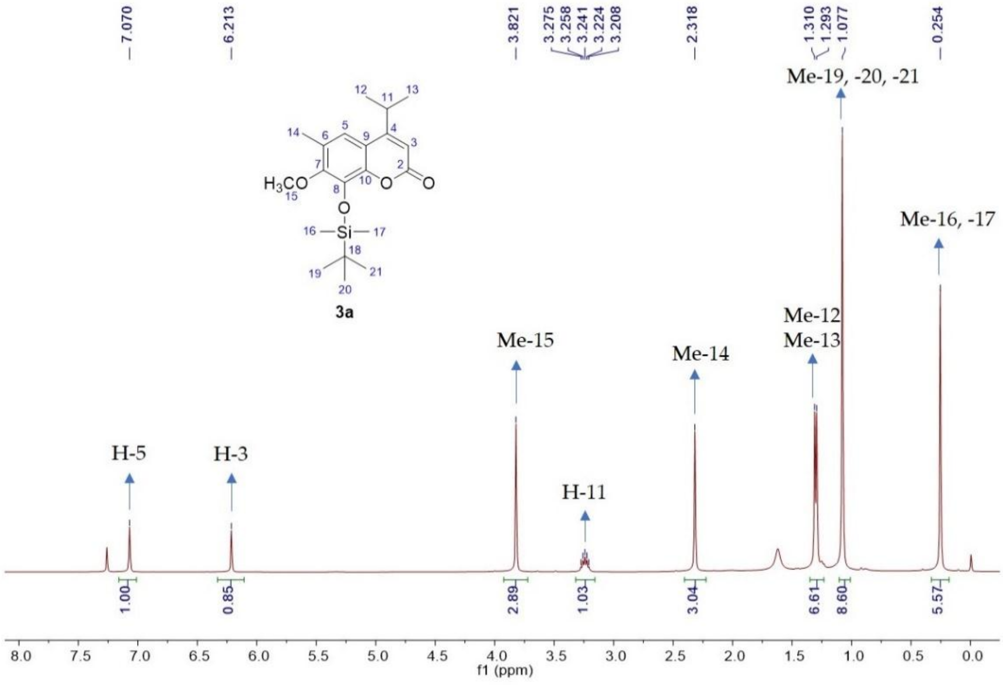

Figure S24. ^{1}H NMR spectrum of compound **3a** recorded in $CDCl_3$ at 400 MHz.

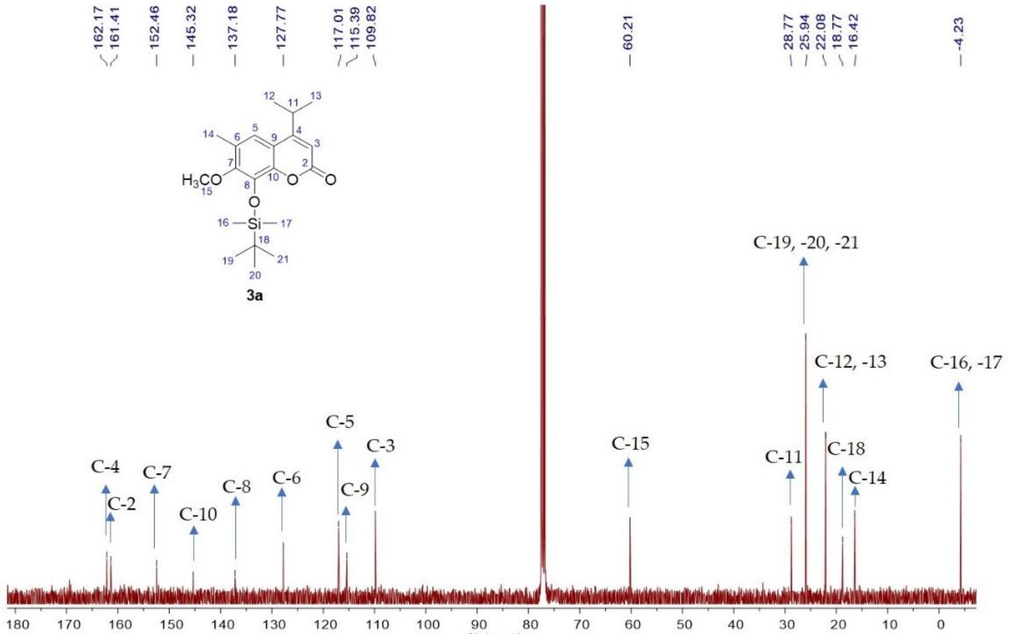

Figure S25. ^{13}C NMR spectrum of compound **3a** recorded in $CDCl_3$ at 100 MHz.

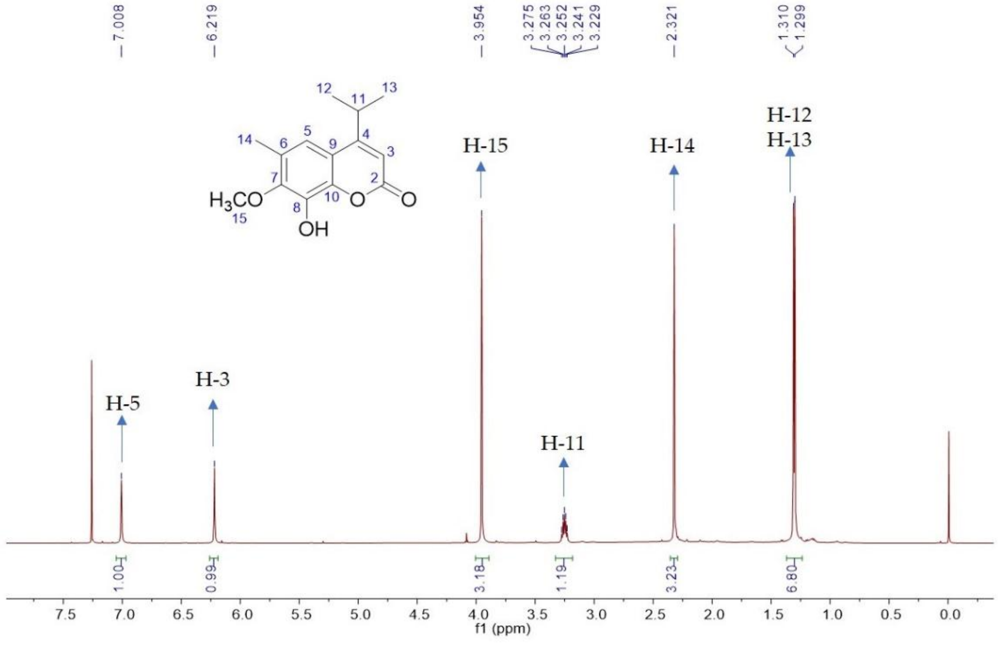

Figure S26. ^{1}H NMR spectrum of compound **3** recorded in $CDCl_3$ at 600 MHz.

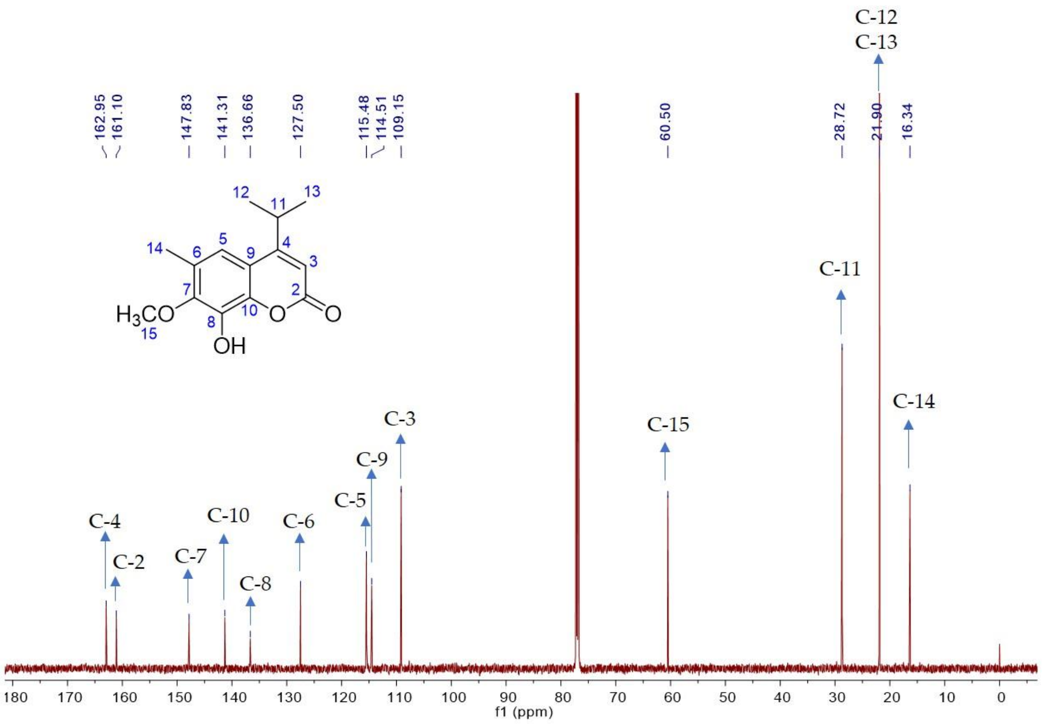

Figure S27. ^{13}C NMR spectrum of compound **3** recorded in $CDCl_3$ at 150 MHz.

^{1}H NMR and ^{13}C NMR spectral data of compounds **4-8:**

vitamin E quinone (**4**): ^{1}H NMR (400 MHz, $CDCl_3$): δ_H 2.54 (2H, m, H-4), 2.03 (3H, s, Me-5a), 2.00 (6H, s, Me-7a, 8a), 1.49 (4H, m, H-3, 1′), 1.23 (3H, s, Me-2a), 1.45~1.00 (19H, m, methines and methylenes), 0.86 (9H, d, *J* = 7.0 Hz, H-4′a, 8′a, 12′a), 0.83 (3H, d, *J* = 7.0 Hz, H-13′); ^{13}C NMR (100 MHz, $CDCl_3$): δ_C 187.8 (C, C-6 or C-9), 187.4 (C, C-6 or C-9), 144.6 (C, C-10), 140.7 (C, C-5), 140.6 (C, C-8), 140.3 (C, C-7), 72.7 (C, C-2), 42.4 (CH_2, C-1′), 40.4 (CH_2, C-3), 39.5 (CH_2, C-11′), 37.8 (CH_2, C-3′), 37.6 ×2 (CH_2, C-7′, 5′), 37.4 (CH_2, C-9′), 32.94 (CH, C-8′), 32.91(CH, C-4′), 28.1 (CH, C-12′), 26.7 (CH_3, Me-2a), 24.9 (CH_2, C-10′), 24.6 (CH_2, C-6′), 22.9 (CH_3, Me-12′a), 22.4 (CH_3, Me-13′), 21.6 (CH_2, C-2′), 21.5 (CH_2, C-4), 19.89 (CH_3, Me-4′a), 19.85 (CH_3, Me-8′a), 12.5 (CH_3, Me-8a), 12.4 (CH_3, Me-7a), 12.1 (CH_3, Me-5a).

albicanol (**5**) : ^{1}H NMR (400 MHz, $CDCl_3$): δ_H 4.94 (1H, br s, H-12a), 4.64 (1H, br s, H-12b), 3.79 (2H, m, H-11), 2.42 (1H, ddd, *J* = 12.9, 4.4, 2.4 Hz, H-7a), 2.04 (1H, m, H-7b), 1.97 (1H, m, H-9), 1.34 (1H, dd, *J* = 12.9, 4.3 Hz, H-5), 1.76 ~ 1.00 (8H, m, H-1, 2, 3, 6), 0.87 (3H, s, Me-15), 0.80 (3H, s, Me-14), 0.72 (3H, s, Me-13); ^{13}C NMR (100 MHz, $CDCl_3$): δ_C 148.0 (C, C-8), 106.4 (CH_2, C-12), 59.3 (CH, C-9), 58.9 (CH_2, C-11), 55.3 (CH, C-5), 42.1 (CH_2, C-3), 39.2 (CH_2, C-7), 39.2 (C, C-10), 38.0 (CH_2, C-1), 33.8 (CH_3, Me-14), 33.6 (C, C-4), 24.4 (CH_2, C-6), 21.9 (CH_3, Me-15), 19.4 (CH_2, C-2), 15.4 (CH_3, Me-13).

2',4'-dihydroxy-6'-methoxy-3',5'-dimethylchalcone (**6**): ^{1}H NMR (400 MHz, $CDCl_3$): δ_H 13.59

(1H, s, OH-6), 5.48 (1H, br s, OH-4), 3.72 (3H, s, OMe-2), 2.69 (3H, s, 1-CO<u>CH3</u>), 2.13 (3H, s, 3-Me or 5-Me), 2.09 (3H, s, 3-Me or 5-Me); ^{13}C NMR (100 MHz, $CDCl_3$): δ_C 203.84 (C, 1-<u>CO</u>CH3), 161.70 (C, C-4 or C-6), 159.39 (C, C-4 or C-6), 143.02 (C, C-2), 109.08 (C, C-3), 108.64 (C, C-5), 106.55 (C, C-1), 61.86 (CH_3, OMe-2), 31.39 (CH_3, 1-CO<u>CH3</u>), 8.76 (CH_3, 3-Me or 5-Me), 7.61 (CH_3, 3-Me or 5-Me); negative ESI-MS: *m*/*z* 209 $[M\text{-}H]^-$, *m*/*z* 419 $[2M\text{-}H]^-$.

norflavesone (**7**): ^{1}H NMR (400 MHz, $CDCl_3$): δ_H 3.94 (1H, m, H-8), 1.88 (3H, s, Me-13), 1.38 (6H, s, Me-11, 12), 1.12 (6H, d, *J* = 6.6 Hz, Me-9, 10); ^{13}C NMR (100 MHz, $CDCl_3$): δ_C 208.3 (C, C-7), 197.1 (C, C-1), 190.0 (C, C-5), 173.3 (C, C-3), 104.8 (C, C-6), 103.1 (C, C-4), 35.7 (CH, C-8), 24.7 × 2 (CH_3, C-11, 12), 19.1 × 2 (CH_3, C-9, 10), 7.0 (CH_3, C-13); ESI-MS: *m*/*z* 237$[M - H]^-$.

aspidinol (**8**): ^{1}H NMR (600 MHz, $CDCl_3$): δ_H 5.95 (1H, s, H-5), 3.82 (3H, s, MeO-4), 3.06 (2H, t, *J* = 7.4 Hz, H-2′), 2.01 (3H, s, Me-3), 1.72 (2H, m, H-3′), 0.99 (3H, *J* = 7.4 Hz, H-4′); ; ^{13}C NMR (150 MHz, $CDCl_3$): δ_C 206.3 (C-1′), 163.2 (C-2), 160.9 (C-4), 159.8 (C-6), 104.8 (C-3), 103.2 (C-1), 91.6 (C-5), 55.8 (MeO-4), 46.3 (C-2′), 18.3 (C-3′), 14.2 (Me-4′), 7.3 (Me-3). ESI-MS: *m*/*z* 223 $[M - H]^-$.
